# Mapping the metabolic responses to oxaliplatin-based chemotherapy with *in vivo* spatiotemporal metabolomics

**DOI:** 10.1016/j.jpha.2023.08.001

**Published:** 2023-08-09

**Authors:** Mariola Olkowicz, Khaled Ramadan, Hernando Rosales-Solano, Miao Yu, Aizhou Wang, Marcelo Cypel, Janusz Pawliszyn

**Affiliations:** a Department of Chemistry, University of Waterloo, Waterloo, ON, Canada; b Jagiellonian Centre for Experimental Therapeutics (JCET), Jagiellonian University, Krakow, Poland; c Latner Thoracic Surgery Research Laboratories, Toronto General Hospital Research Institute, University Health Network, Toronto, ON, Canada; d The Jackson Laboratory, JAX Genomic Medicine, Farmington, CT, USA; e Division of Thoracic Surgery, Department of Surgery, University Health Network, University of Toronto, Toronto Lung Transplant Program, Toronto, ON, Canada

**Keywords:** Pulmonary metastases, Colorectal cancer, Adjuvant chemotherapy, In vivo lung chemo-perfusion, Solid-phase microextraction (SPME) microprobes, Spatial metabolomics

## Abstract

Adjuvant chemotherapy improves the survival outlook for patients undergoing operations for lung metastases caused by colorectal cancer (CRC). However, a multidisciplinary approach that evaluates several factors related to patient and tumor characteristics is necessary for managing chemotherapy treatment in metastatic CRC patients with lung disease, as such factors dictate the timing and drug regimen, which may affect treatment response and prognosis. In this study, we explore the potential of spatial metabolomics for evaluating metabolic phenotypes and therapy outcomes during the local delivery of the anticancer drug, oxaliplatin, to the lung. 12 male Yorkshire pigs underwent a 3 h left lung in vivo lung perfusion (IVLP) with various doses of oxaliplatin (7.5, 10, 20, 40, and 80 mg/L), which were administered to the perfusion circuit reservoir as a bolus. Biocompatible solid-phase microextraction (SPME) microprobes were combined with global metabolite profiling to obtain spatiotemporal information about the activity of the drug, determine toxic doses that exceed therapeutic efficacy, and conduct a mechanistic exploration of associated lung injury. Mild and subclinical lung injury was observed at 40 mg/L of oxaliplatin, and significant compromise of the hemodynamic lung function was found at 80 mg/L. This result was associated with massive alterations in metabolic patterns of lung tissue and perfusate, resulting in a total of 139 discriminant compounds. Uncontrolled inflammatory response, abnormalities in energy metabolism, and mitochondrial dysfunction next to accelerated kynurenine and aldosterone production were recognized as distinct features of dysregulated metabolipidome. Spatial pharmacometabolomics may be a promising tool for identifying pathological responses to chemotherapy.

## Highlights

• The potential of in vivo spatiotemporal metabolomics was explored for evaluating the chemotherapy outcome.
• SPME microprobes were employed with global metabolite profiling.
• Metabolic phenotypes were identified during the local delivery of oxaliplatin to the porcine lung.
• Massive alterations in the metabolic patterns were recognized being indicative of acute lung injury (ALI).
• Spatiotemporal metabolomics may improve ALI diagnosis and facilitate adjustments to the chemotherapy regimen.

## Graphical abstract

**Figure:**
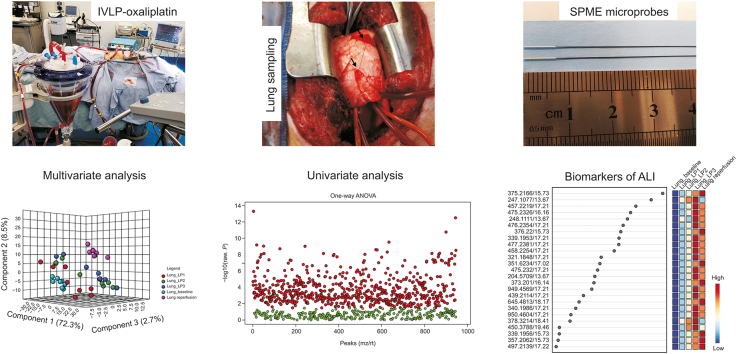

## 1 Introduction

Colorectal cancer (CRC) has emerged as a major public health problem in developing countries, becoming the third most-commonly diagnosed cancer and the second leading cause of death globally after cardiovascular disease [1,2]. Patients with localized CRC typically have a 90% 5-year survival rate; however, a patient's chances of survival decline rapidly as the cancer spreads to other organs. After the liver, the lung is the most common site of metastases in CRC patients, with lung seeding occurring in approximately 10% of patients who have been surgically treated for primary CRC [3]. Pulmonary metastasectomy has been widely accepted as a curative option in the multimodal treatment of metastatic CRC patients [[3], [4], [5]]. The 5-year survival rates of patients who benefited from pulmonary metastasectomy range between 27% and 68% (after complete resection); in contrast, the 5-year prognoses of patients with metastatic CRC who receive only supportive care are less than 5%. Furthermore, adjuvant chemotherapy following the resection of CRC pulmonary metastases may reduce recurrences and improve survival [6,7]. Nonetheless, there remains much debate regarding the best candidates for adjuvant chemotherapy, along with the optimal biological agents and dose regimens, especially in the resection of a single lung metastasis.

Over the past two decades, metabolomics, which aims to comprehensively identify/study and interpret the complex interactions between small molecules in biological systems, has attracted much attention for its potential in (pre)clinical settings [[8], [9], [10]]. In particular, metabolomics enables the discovery of novel biomarkers, which supports the development of early personalized diagnoses, treatments, and dose regimens aimed at preventing the progress of the disease [10,11]. Advances in technology have further bolstered the prominence of omics sciences, particularly metabolomics, as this approach can be employed in a range of (pre)clinical applications, such as identifying diagnostic biomarkers of various diseases, elucidating the disease's mechanisms and the pathophysiology of the target clinical phenotype, discovering novel drug targets, and, ultimately, predicting treatment responses. Along with providing a more precise understanding of individual patient responses to applied therapies, pharmacometabolomic studies have shown promising results in predicting drug efficacy and toxicity [12]. As metabolomics considers individuals as unique combinations of physiological, biochemical, and environmental interactions, the interplay between drug pharmacology and the patient's (patho)physiology could be utilized to optimize pharmacotherapies, thereby creating more personalized options and, thus, advancing precision medicine.

Compared to pharmacogenomics, which uses genetic polymorphisms to predict individual variations in responses to applied treatment, metabolomics offers several advantages that enhance its ability to explain inter-individual variability in drug pharmacokinetics or pharmacodynamics [12]. One such advantage is the ability of metabolomics to provide a direct readout of a patient's current metabolic state, which contains current information related to the cellular activity and potential drug treatment outcomes. Given that the patient's (patho)physiology changes during the stages of disease and pharmacotherapy, it is of paramount importance to consider endogenous metabolites not as static variables in isolation, but rather, as a highly dynamic, time-dependent network [12,13]. Such investigations should examine how drug dosage and temporal changes in drug concentrations impact metabolite network interactions before using metabolite markers to inform the selection of drug dosage regimens in patients.

In metabolomics studies, the experiment design, and especially the selected sampling method, is of the utmost importance [14] and should be carefully configured to capture dynamic fluctuations in the cellular metabolome. Solid-phase microextraction (SPME) is a versatile, non-exhaustive sample-preparation technique that has proven to be suitable for facile and effective analysis of a broad range of compounds in various complex biological matrixes [15]. Multiple SPME formats (e.g., fibers, thin film (blades), dispersed particles, and in-tube) and coatings (extractive phases) have been successfully applied for the determination of diverse analytes in various biological samples, including biofluids, intact tissues, and cell cultures [15,16]. SPME's broad selection of configurations and coating chemistries enables case-specific solutions that aren't possible with other sample-preparation tools. Of the various SPME geometries, fibers and thin films have been applied most frequently to meet the very specific needs of many studies, particularly those in the biomedical field. In terms of global metabolite or lipid profiling, fiber-based SPME provides new pathways for investigating biochemical processes, predominately by enabling the *in vivo* sampling of complex biological systems, thus allowing the capture of unstable metabolites via streamlined metabolism quenching [[17], [18], [19]]. Additionally, since *in vivo* SPME enables nonlethal and/or non-destructive sampling, it can be deployed to perform multiple samplings from the same animal or sample in time-course-based metabolomics studies, thereby providing a true metabolic snapshot of the subject, including the elusive/unstable fraction of metabolites, which is not captured when using *ex vivo* approaches. Furthermore, SPME-based fast-profiling applications are of particular interest in hospital diagnostic facilities. Here, combining SPME with shotgun lipidomics strategies may enable the development of state-of-the-art platforms for tissue analysis, as dysregulation in the lipid metabolism has been widely recognized as one of the most prominent metabolic alterations in cancer patients [19,20]. The intrinsic features of directly coupling SPME with analytical detection systems have the potential to yield significantly shorter analytical turnaround times and higher sensitivity, which may ultimately provide intriguing alternatives to existing point-of-care technologies. Further, the better spatial resolution provided by smaller SPME probes may enable the monitoring of biological alterations at even the single-cell level, without causing any apparent disturbances in the living system [15,19,21]. Overall, SPME is a very promising technology with myriad potential future applications that can provide powerful insights into the biological roles of molecules and processes in influencing the state of living organisms.

Building on our previous work [[22], [23], [24]] and expanding on other metabolomics studies featuring the development of miniaturized microsampling methods [18,25], the present work documents a comprehensive approach that enables the detailed pharmacometabolomic profiling of the lung during *in vivo* lung chemo-perfusion in pre-clinical settings. This work aims to provide new insights into drug (oxaliplatin) activity and toxicity and associated disturbances in metabolomic patterns in order to identify metabolic markers that can be used to determine treatment outcomes.

## 2 Materials and methods

### 2.1 Experimental design and statistical rationale

In total, 12 male Yorkshire pigs weighing an average of 38 ± 5 kg underwent a 3 h *in vivo* lung perfusion (IVLP) procedure, followed by 72 h of treatment in which final endpoint functional and biochemical assessments were performed. After these assessments were completed, the pigs were euthanized via exsanguination under anesthesia. An accelerated titration dose-escalation study design was employed wherein oxaliplatin (OxPt) doses were sequentially doubled if no apparent toxicity was observed during the previous case. Toxicity was assessed using computed tomography (CT) imaging and histology to verify the preservation of lung function or the absence of signs of severe acute lung injury. OxPt (Pfizer, Kirkland, QC, Canada) was injected into the perfusion circuit reservoir as a bolus once full perfusion flow had been established. The chemotherapy dose was set to achieve a target concentration in the perfusion circuit, and was calculated based on standard doses of OxPt used in clinical regimens. Accordingly, the starting dose of 10 mg/L was selected and sequentially doubled until reaching 80 mg/L, where clinically significant lung injury was observed. More details regarding the IVLP procedure, perfusion circuit, the composition of the priming solution, and protective perfusion/ventilation strategy can be found in our recent work [23].

The one-dimensional liquid chromatography-mass spectrometry (1D-LC-MS)-based profiling of global metabolites or lipid species was conducted using seven 72 h porcine survival cases wherein the left lung was treated with OxPt at doses of 7.5, 10, 20, 40, and 80 mg/L. Samples were collected at baseline (pre-perfusion), hourly during IVLP, and at reperfusion using SPME fibers coated with polyacrylonitrile (PAN)-based extraction phases containing C_18_ or mixed-mode (MM; C_8_+benzenesulfonic acid) functionalities. For tissue measurements, 2 MM-coated microprobes (280 μm in diameter) were inserted into 2 different lung regions—one in the upper lobe, and one next to the lingula—at each time point. Perfusate sampling was conducted using 3 MM and 3 C_18_ fibers, while plasma sampling was performed using 3 MM devices. The general microsampling procedure consisted of the following steps: (a) preconditioning the coating material in a MeOH/H_2_O solution (1:1, *V*/*V*) to ensure it was ready to interact with the sample matrix; (b) a 20 min static extraction by directly immersing the microdevices into the complex matrix (the lung, perfusate or plasma); (c) a quick washing step with water (for 5 s) to remove any materials loosely attached to the coating surface; and (d) a solvent desorption step that had been optimized to guarantee minimum carryover and maximum compatibility with the employed chromatographic conditions: for the MM probes acetonitrile (ACN)/H_2_O (8:2, *V*/*V*), while for the C_18_ probes MeOH/isopropanol (IPA)/H_2_O (45:45:10, *V*/*V*/*V*). The same processing procedure was used to prepare blank fibers (extraction blanks), which served as negative controls. In total, 295 replicates (70 lungs, 168 perfusates and 57 plasma samples) were collected and analyzed via metabolomics or lipidomics. The order in which the samples were subjected to LC/MS analysis was randomized. Pooled quality control (QC) samples representing the sample matrix and metabolite composition were prepared by pooling equal volumes of each biological/technical replicate and analyzed every 10 injections during the metabolomic and lipidomic analyses.

The experimental protocol was approved by the Animal Care Research Committee at the Toronto General Hospital Research Institute (University Health Network (UHN); Toronto, ON, Canada) and the University of Waterloo's Research Ethics Board (Approval number: #40,573), and all procedures conformed to the NIH guidelines (Guide for the Care and Use of Laboratory Animals).

### 2.2 LC-MS analysis

Extracts were analyzed on a pentafluorophenyl (PFP) stationary phase (MM microprobes/metabolomics) or a C_18_ column (C_18_ microprobes/lipidomics) using a Vanquish Flex UHPLC connected to an Exactive Orbitrap High-Resolution, Accurate-Mass (HRAM) mass spectrometer (Thermo Scientific, Waltham, MA, USA) equipped with heated electrospray ionization (HESI) source. Since selecting an appropriate SPME extraction phase can enable balanced analyte coverage [15,19,26], two analytical protocols for capturing polar and semi-polar compounds (MM probes), along with non-polar ones (C_18_ probes), were employed to bolster the amount of information obtained about the studied system. In addition, although the PFP and C_18_ phases offered similar reversed-phase (RP) selectivity, the PFP phase outperformed the C_18_ phase in terms of the number of polar and moderately hydrophobic metabolites retained.

In line with the above, for metabolomic investigations, the analytes were separated on a Discovery HS F5 column (100 mm × 2.1 mm, 3 μm particle size; Supelco, Oakville, ON, Canada) maintained at 25 °C. Metabolites were eluted from the column at 300 μL/min using a two-component (binary) mobile phase consisting of deionized water (A) and ACN (B), with 0.1% formic acid (ve+ mode) or 1 mM acetic acid (ve– mode). The gradient elution was as follows: 0% B for 3 min, 0%–90% B in 22 min, hold for 9 min, then back to 0% B in 1 min, and re-equilibration of the column for 5 min. In turn, chromatographic separation of lipid species in the extracts collected was performed at 55 °C with an XSelect CSH C_18_ column (75 mm × 2.1 mm, 3.5 μm; Waters, Mississauga, ON, Canada) using a two-solvent system composed of solvent A (MeOH:H_2_O (40:60, *V*/*V*) with 10 mM ammonium acetate and 1 mM acetic acid (ve+ mode) or with 0.02% acetic acid (ve– mode)) and solvent B (IPA:MeOH (90:10, *V*/*V*) with 10 mM ammonium acetate and 1 mM acetic acid (ve+ mode) or with 0.02% acetic acid (ve– mode)). The samples were kept in a vial tray which was set at 4 °C and the flow rate was maintained at 300 μL/min. Chromatographic separation was achieved in 27 min run time with the following gradient: 0–2 min, 20% B; 2–3 min, 20%–30% B; 3–13.5 min, 30%–80% B; 13.5–18.5 min, 80%–85% B; 18.5–20 min, 85%–95% B; 20–21.5 min, 95% B; 21.5–27 min, 20% B.

The Exactive Orbitrap acquired full scan data using a mass resolution of 50,000 in both modes with a mass range of 100–1000 *m*/*z*. The LC-MS system run parameter details are available in Table S1.

To assess instrument performance and the quality of data obtained in the metabolomics or lipidomics studies, QC samples composed of reference standards, replicate extracted samples, pooled QC samples comprised of a mixture of aliquots taken from every sample in the study, and process blanks were analyzed alongside the batch [27,28]. The post-hoc chemical identity of significantly altered and biologically relevant compounds was verified by applying tandem mass spectrometry (MS/MS) data acquisition to a set of pooled QC samples.

### 2.3 Data processing and statistical analysis

All data analyses were performed within the R Studio environment using custom scripts written in the R programming language. Briefly, raw data files acquired via Thermo Xcalibur software (ver. 3.0.63) were converted to mzXML format using MSConvert (ProteoWizard 3.0) [29]. The processing parameters for XCMS online were optimized by using the pooled QC samples with the isotopologue parameters optimization (IPO) tool to ensure that biological or inter-individual variability did not affect the selection of parameters [30]. Since the IPO package employs an upper and lower level for each parameter to be adjusted (before ultimately calculating the center point), an iterative process was used until the optimized parameter setting was determined. Subsequently, the adjusted parameters were applied to all samples for final peak picking, retention time correction, and peak grouping. Finally, the CAMERA 1.43.2 package was applied to detect isotopologues and adducts. The analysis of the data yielded a two-dimensional table (matrix) containing a list of features and signal intensities for each sample set and ESI ionization mode. These matrices were then filtered to remove artefacts present in the process blank samples, as well as peaks demonstrating significant variability. Additionally, the GlobalStd algorithm, implemented in a PMD package, was applied to reduce redundant peaks in the metabolomics data profile [31]. The curated data files (peak tables) were subsequently imported to MetaboAnalyst 5.0 web-based software for further statistical and pathway enrichment analysis [32].

Multivariate statistical analyses, principal component analysis (PCA), and partial least squares-discriminant analysis (PLS-DA) were carried out after log transformation and pareto scaling. Since PLS-DA is a supervised method, the accuracy of the generated PLS-DA models was verified via leave-one-out cross-validation (LOOCV) and the reporting of the R2 and Q2 values, which represented the estimates of the goodness-of-fit and predictive ability for the models, respectively. A 1000-times permutation test was subsequently used to evaluate the robustness of the PLS-DA models and to assess the risk of overfitting them. The variable importance in projection (VIP) scores were also calculated to evaluate how metabolic variables contributed to the classification, with VIP scores ≥1.0 being considered significant. The significance of altered metabolites was subsequently investigated via one-way analysis of variance (ANOVA), along with false discovery rate (FDR) corrections. Metabolites or lipids with VIP scores ≥1.0, ANOVA test *P*-values <0.05, an adjusted FDR <0.01, and a fold change threshold of 1.5 were selected as important analytes for group classification and further biochemical pathway analysis.

### 2.4 Compound identification and pathway analysis

The xMSannotator Integrative Scoring Algorithm incorporated into an R package was employed to retrieve the metabolite identifications (IDs) and names from three publicly available databases [33]. To annotate ions for possible chemical identity, multistage clustering was performed in which metabolic pathway associations were recognized/used along with their intensity profiles, retention time characteristics, mass defect, and isotope/adduct patterns. This enabled confidence levels to be assigned to the annotation results. The Kyoto Encyclopedia of Genes and Genomes (KEGG), Human Metabolome Database (HMDB), and LIPID MAPS Structure Database (LMSD) served as database references for querying. As a result, the software identified compounds by categorizing annotations based on database matches into different confidence levels, thus allowing the prioritization of analytes for further validation efforts. Whenever possible, metabolites or lipid species were identified/confirmed based on the analysis of authentic standards or searching the MassBank, MoNA, or LipidBlast libraries for tandem mass spectral data (MS/MS) using the NIST MS Search (v. 2.3) or MS-FINDER (v. 3.52) software tools [[34], [35], [36]].

Metabolic pathway enrichment was performed using the metabolite set enrichment analysis (MSEA) module and the online KEGG/HMDB database, both integrated into MetaboAnalyst 5.0. The pathway impact scores (generated via MSEA analysis) represented the potential of each dysregulated pathway in the study. The top 25 enriched/affected pathways were identified and constructed according to the potential functional analysis.

## 3 Results

### 3.1 Exploring and probing metabolism with in vivo SPME

To examine metabolic alterations during the local administration of OxPt-based therapy, and to identify potential biomarkers, which can potentially improve the prediction of acute lung injury (ALI) in high-risk (pre)clinical conditions, 70 tissue samples and 168 perfusate samples were collected during low-dose (7.5−20 mg/L) and high-dose (40−80 mg/L) treatment regimens. Global metabolomics and lipidomics were applied to profile the metabolic changes caused by the chemotherapy. The samples were examined using two different analytical platforms (PFP-HRAM/MS(Orbitrap) for metabolomics and CSH-HRAM/MS(Orbitrap) for lipidomics) and two different ionization modes (positive and negative) to increase coverage to include analytes spanning diverse chemical classes (Table S1). This instrumental analysis was followed by analyte identification, multivariate/univariate statistical analysis, and interpretation, which resulted in the identification of 49 potential discriminant metabolites/lipids in the tissue extracts and 90 differentiating variables in the perfusates. In addition, PLS-DA performed on the analytical data showed the clustering of all QCs on the obtained plots, indicating stable and consistent system performance throughout the entire analytical run (Figs. S1–S3).

**Fig. 1:**
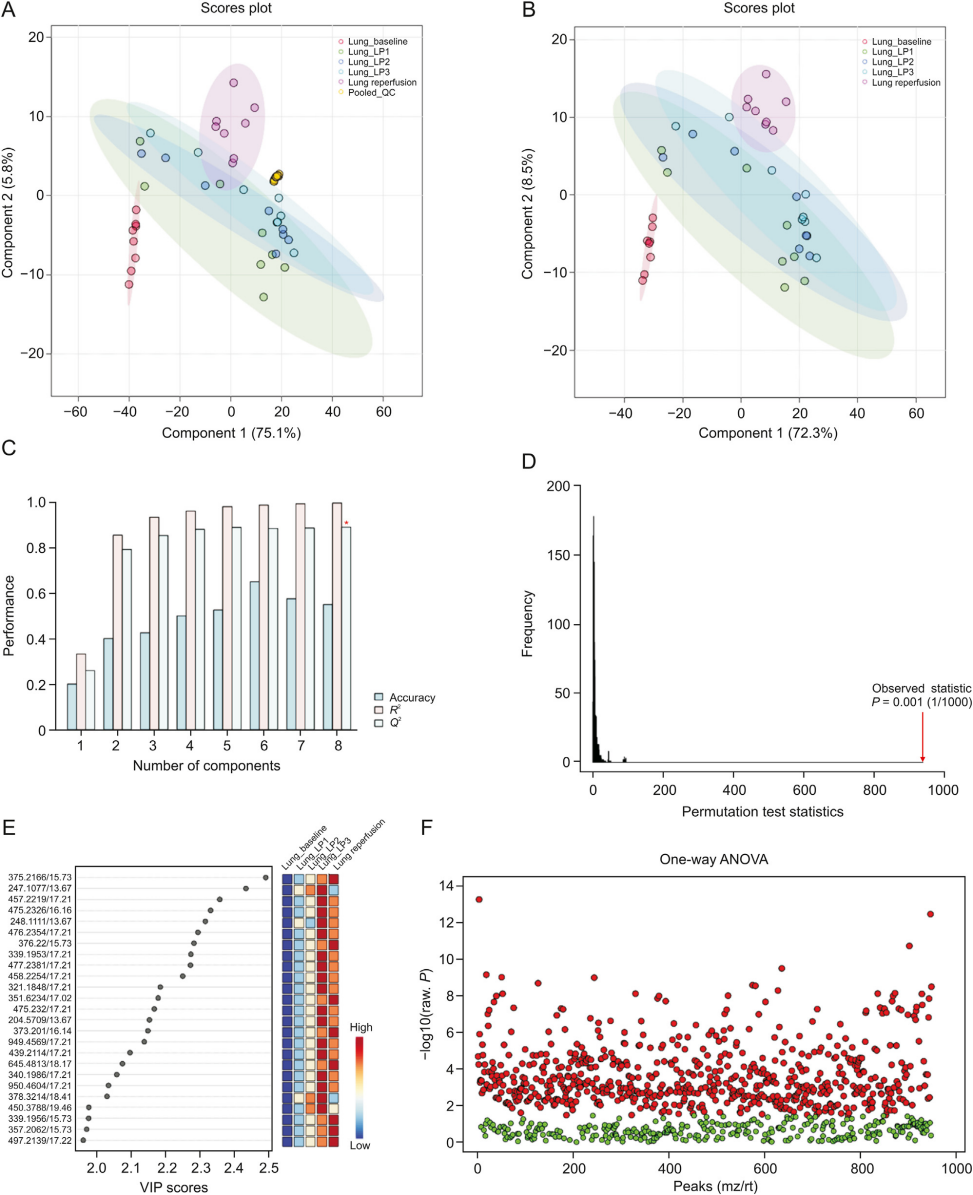
Partial least squares-discriminant analysis (PLS-DA) along with univariate one-way analysis of variance (ANOVA) based on lung metabolites determined via solid phase microextraction-liquid chromatography/mass spectrometry using positive ion mode (SPME-ESI (+)-LC/MS). (A, B) Two-dimensional score plots showing clustering and separation between metabolomic profiles corresponding to various sampling time-points during *in vivo* lung chemo-perfusion. The pooled quality control (QC) sample sets are segregated in a tight cluster in the centre of the PLS-DA score plot, which indicates the excellent robustness of the developed SPME-LC/MS method. (C) PLS-DA classification performance using increasing numbers of latent variables. Leave-one-out cross-validation (LOOCV) was applied to evaluate the level of overfitting for the PLS-DA model. The attained *Q*^2^ value of 0.89 indicates that the model provides good predictability for the dataset. The red star depicts that the best model was obtained using 8 variables. (D) Permutation test results based on 1,000 iterations. (E) The top 25 most discriminant metabolite features were revealed by variance in projection (VIP) analysis. (F) The attained VIP metabolites were further verified via univariate one-way ANOVA analysis. Lung_LP1/LP2/LP3: samples collected at the 1st, 2nd, and 3rd h of *in vivo* lung perfusion (IVLP); mz/rt: feature/individual ion with a unique mass-to-charge ratio and a unique retention time.

**Fig. 2:**
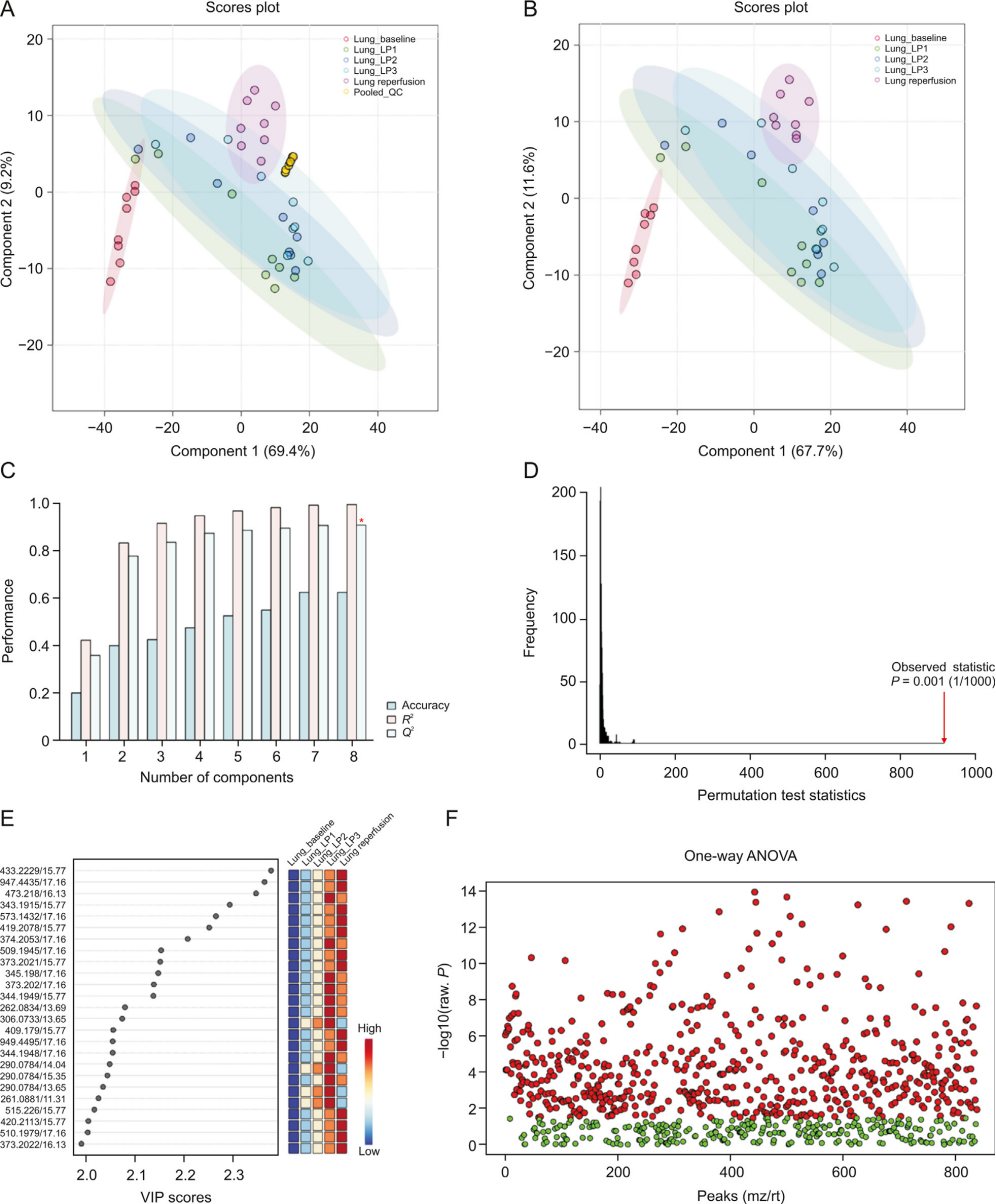
Partial least squares-discriminant analysis (PLS-DA) along with univariate one-way analysis of variance (ANOVA) based on lung metabolites determined via solid phase microextraction-liquid chromatography/mass spectrometry using negative ion mode (SPME-ESI (−)-LC/MS). (A, B) Multivariate analysis of metabolomic data using PLS-DA resulted in the distinct separation of metabolic features among pre-perfusion, *in vivo* lung perfusion (IVLP), and reperfusion events. Tightly clustered (vs. the biological replicates) pooled quality control (QC) samples created by mixing numerous representative biological samples, which supports the assessment of data quality. (C) Leave-one-out cross-validation (LOOCV) confirmed that the PLS-DA models were able to accurately discriminate between the pre-perfusion or blood reperfusion period and the IVLP event. (D) Random permutation test examining the robustness of the PLS-DA model (based on separation distance). (E) The top 25 differentiating metabolite features revealed by variable importance in projection (VIP) analysis. A VIP ≥1.0 was considered statistically significant. (F) VIP results were verified by univariate ANOVA analysis. Lung_LP1/LP2/LP3: samples collected at the 1st, 2nd, and 3rd hour of *in vivo* lung perfusion (IVLP); mz/rt: feature/individual ion with a unique mass-to-charge ratio and a unique retention time.

**Fig. 3:**
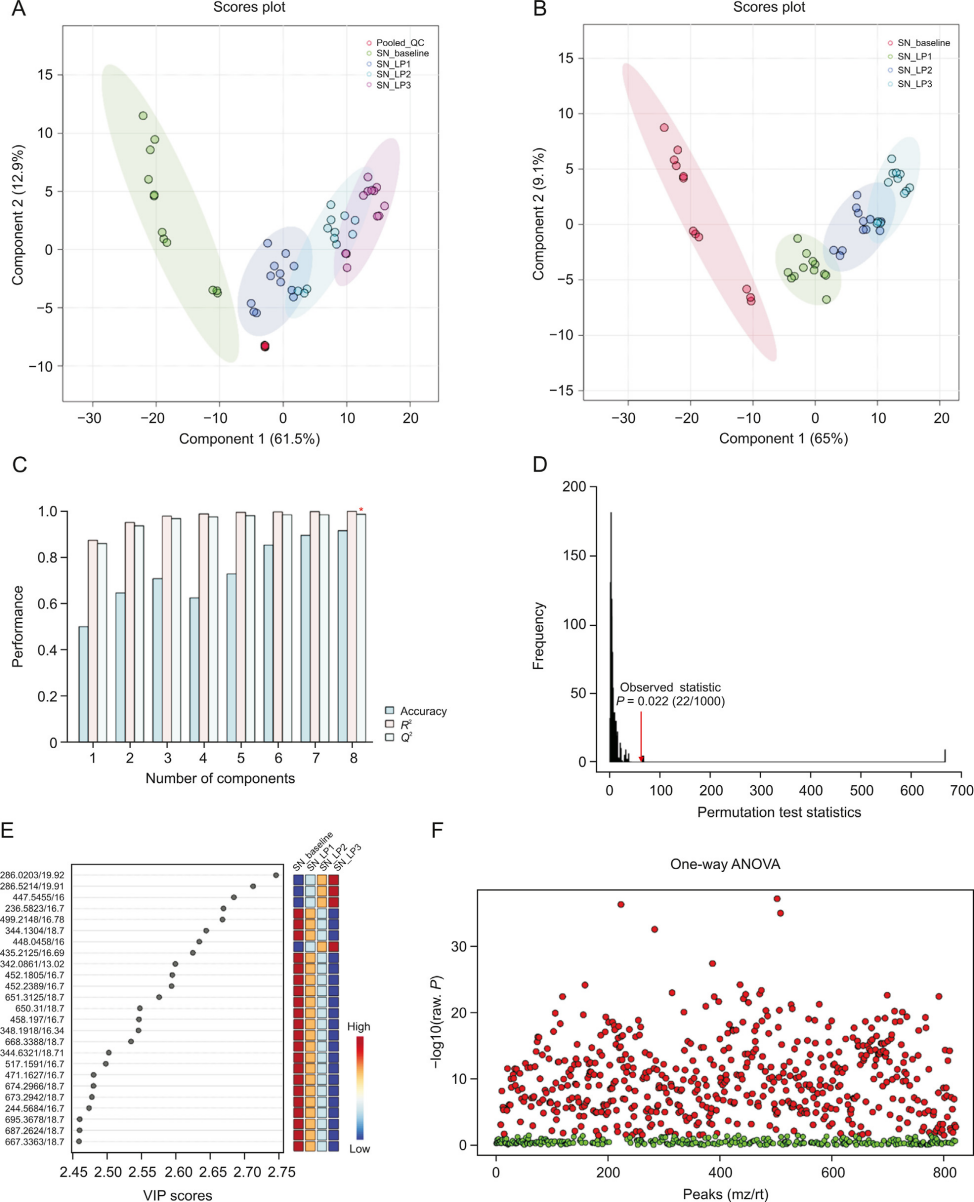
Partial least squares-discriminant analysis (PLS-DA) and cross-validation analysis along with univariateone-way analysis of variance (ANOVA)of SPME-ESI (+)-LC/MS metabolomics data for the extracts obtained using mixed-mode-based perfusate sampling*.* (A, B) PLS-DA projections in two dimensions with a 95% confidence interval for each group. (C) PLS-DA classification using different numbers of components. The red star indicates the best classifier with 8 components involved. The classification performance values for accuracy, goodness of fit (R2), and predictive ability (Q2) for the top 8 components, were 0.92, 1.0, and 0.99, respectively. (D) Permutation test statistics for 1000 iterations with observed statistics at *P* = 0.02. (E) Metabolite features showing a variable importance in projection (VIP) score >2.0 in the PLS-DA analysis. (F) An adjusted *P*-value (FDR) cut-off of 0.01 was applied for the one-way ANOVAs. SN_baseline: samples collected before oxaliplatin administration; SN_LP1/LP2/LP3: samples collected at the 1st, 2nd, and 3rd hour of *in vivo* lung perfusion (IVLP); QC: quality control samples; mz/rt: feature/individual ion with a unique mass-to-charge ratio and a unique retention time.

**Fig. 4:**
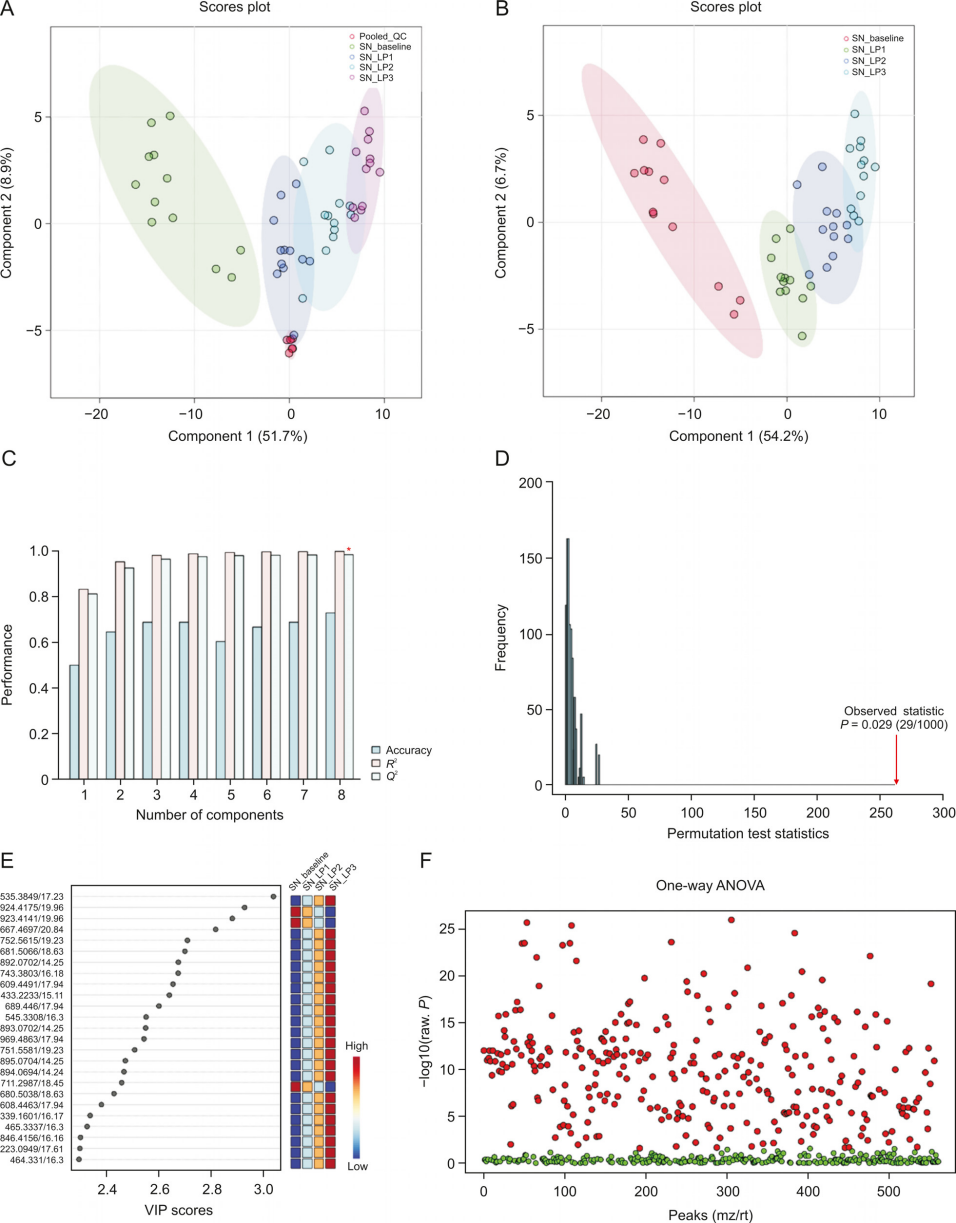
Partial least squares-discriminant analysis (PLS-DA) and cross-validation analysis along with univariateone-way analysis of variance (ANOVA) of SPME-ESI (−)-LC/MS metabolomics data for the extracts obtained using mixed-mode-based perfusate sampling. (A, B) Score plots for the first two components of the PLS-DA analysis. As can be seen, the quality control (QC) samples made by pooling small aliquots of the studied samples are clustered together in the PLS-DA plot, thus confirming the analytical platform's stability throughout the run. (C) Evaluation of PLS-DA classification performance using increasing numbers of latent variables. Leave-one-out cross-validation (LOOCV) indicated that the model shown with the red asterisk was best classified by 8 components. The classification performance values for accuracy, goodness of fit (*R*^2^), and predictive ability (Q^2^) for the top 8 components were 0.73, 1.0, and 0.98, respectively. (D) PLS-DA model validation via permutation tests based on separation distance classifier (statistical validation obtained via 1000-times permutation tests). The resultant *P*-value was *P* = 0.029. (E) Metabolic features ranked by variable importance in projection (VIP). VIP threshold ≥1.0. (F) VIP results verified by univariate ANOVA analysis. SN_baseline: samples collected before oxaliplatin administration; SN_LP1/LP2/LP3: samples collected at the 1st, 2nd, and 3rd h of *in vivo* lung perfusion (IVLP); mz/rt: feature/individual ion with a unique mass-to-charge ratio and a unique retention time.

**Fig. 5:**
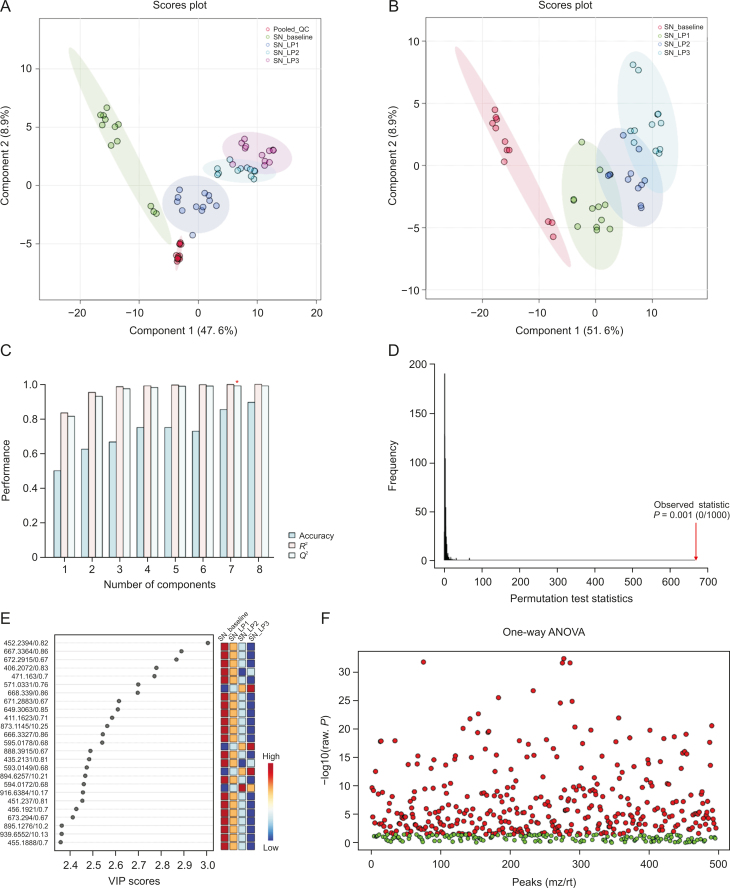
Score plots for partial least squares-discriminant analysis (PLS-DA), cross-model validation, and permutation testing to validate the classification models based on perfusate lipidomic analysis data (SPME-RP/LC-MS ESI (+) mode). (A, B) PLS-DA models (two dimensional) with a 95% confidence interval for each group. (C) Estimate of the model's predictive ability, which was calculated via cross-validation (CV) and plotting *R*^2^, *Q*^2^, and the accuracy values. Class discrimination performance was measured via the classification accuracy (0.85), *R*^2^ (1), and *Q*^2^ (0.99) parameters. (D) Permutation test statistics for 1,000 permutations with observed statistics at *P* < 0.001. (E) Variable importance in projection (VIP) scores with the top differential metabolites/lipids. (F) VIP results verified by one-way analysis of variance (ANOVA) testing. SN_baseline: samples collected before oxaliplatin administration; SN_LP1/LP2/LP3: samples collected at the 1st, 2nd, and 3rd hour of *in vivo* lung perfusion (IVLP); QC: quality control samples; mz/rt: feature/individual ion with a unique mass-to-charge ratio and a unique retention time.

**Fig. 6:**
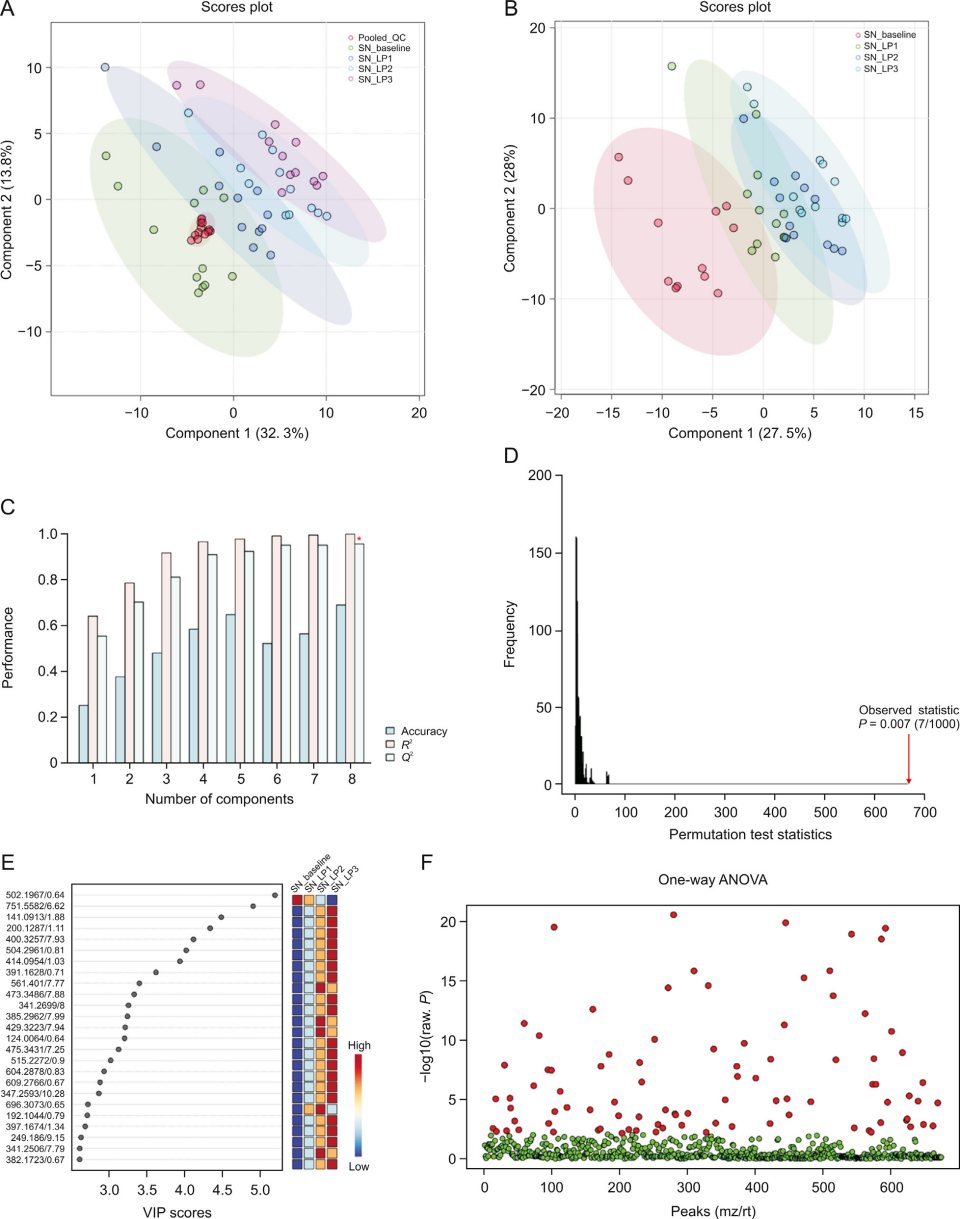
Score plots for the partial least squares-discriminant analysis (PLS-DA), cross-model validation, and permutation testing to validate the classification models based on perfusate lipidomic analysis data (SPME-RP/LC-MS ESI (−) mode). (A, B) 2D PLS-DA plots. All pooled quality control (QC) samples were tightly clustered in the centre of the PLS-DA scores plot, indicating the SPME-LC/MS method's good reproducibility and the absence of bias introduced during dataset processing. (C) The classification performance (of the model) was assessed by accuracy (0.69), goodness of fit (1.0), and predictive ability (0.95) for the top eight components. (D) Permutation test for the generated model (*P* = 0.007). (E) Variable importance in projection (VIP) score plot based on two components from the PLS-DA. (F) One-way analysis of variance (ANOVA) test with multiple testing correction using false discovery rate (FDR). SN_baseline: samples collected before oxaliplatin administration; SN_LP1/LP2/LP3: samples collected at the 1st, 2nd, and 3rd h of *in vivo* lung perfusion (IVLP); mz/rt: feature/individual ion with a unique mass-to-charge ratio and a unique retention time.

Visual inspection of the data revealed profound metabolic differences in the most abundant metabolites and lipid species in the high-dose group; however, to confirm these changes and evaluate other subtle metabolic alterations, detailed multivariate statistical analysis was conducted by comparing the normalized spectral features and constructing the score plots retrieved from the PLS-DA analysis. These plots are shown in Figs. 1A and B (ESI (+)), as well as Figs. 2A and B (ESI (−)). The score plots clearly showed exquisite separation among the samples collected at various time-points (pre-perfusion, 3h lung perfusion, reperfusion) and clustering of the samples in each study group, indicating that the study groups were distinct in terms of their metabolic profiles. This result was further corroborated by the significantly higher (*R*^2^, *Q*^2^ > 0.9) validation parameters of the PLS-DA models (Figs. 1C and 2C), suggesting the discriminatory models possessed good predictive power. A permutation test based on 1000 iterations was also performed along with cross validation, resulting in an empirical *P* < 0.05 (Figs. 1D and 2D). Furthermore, metabolites or lipids with a VIP score ≥1, an ANOVA test *P*-value <0.05, an adjusted FDR <0.01, and a fold change threshold of 1.5 were selected as important species for group classification (Figs. 1E, 1F, 2E, and 2F, and Table S2). The differentiating metabolites/lipids included amino acids (AAs), dipeptides, nucleotides and nucleosides, fatty acids (FAs) conjugates, lipids, steroids, tricarboxylic acid cycle (TCA) metabolites, and β/ω-oxidation intermediates. Similarly, the 2D and 3D PLS-DA plots showed the clear separation of metabolomic features among low-dose OxPt-treated subjects (Figs. S1 and S4), although the number of dysregulated species was less pronounced (vs. the high-dose group) next to the magnitude of this dysregulation. Next, the discriminatory potential of metabolic features in the perfusate samples from the high-dose group was identified by indexing the variable importance in projection (VIP) scores based on the PLS-DA analysis and ANOVA results (Fig. 3, Fig. 4, Fig. 5, Fig. 6). The sample clustering and group separation among the four studied conditions (baseline vs. 1–3 h IVLP perfusion) was clearly visible in the 2D PLS-DA evident (with model validation parameters, *R*^2^ and *Q*^2^ > 0.9). 47 metabolites distinguishing the IVLP condition from the pre-perfusion condition were identified, including AAs, various FAs, steroids, eicosanoids, central carbon metabolism intermediates, and diverse lipid mediators (Fig. 3, Fig. 4, and Table S3). Similarly, lipidomic investigations identified 43 dysregulated lipid species in the perfusate samples (Fig. 5, Fig. 6, and Table S4). As shown in Figs. 5A and B, the PLS-DA model generated for positive ion mode provided complete separation between the samples before and after OxPt treatment. While the PLS-DA models for negative ion mode ESI (–) did not produce efficient separation between the groups, a clear tendency for separation was evident in each comparison (Figs. 6A and B). This separation revealed significant alterations in the levels of glycerophospholipids, sphingolipids, oxylipins, polyunsaturated fatty acids (PUFAs), and acylcarnitines, as well as low-molecular-weight compounds, such as hydrophobic AAs and porphyrins. Classification PLS-DA models were also constructed for the low-dose group, however the discrimination trends over the various stages of the lung perfusion were less evident (Figs. S5 and S6). Interestingly, partial overlapping of altered metabolites/pathways was observed in both the tissue and perfusate samples, demonstrating that each matrix presented a distinct analyte pattern.

Global metabolic profiling was also conducted on blood plasma samples collected during IVLP. Here, the resultant score plots showed a trend towards clustering among samples collected at specific time points of the procedure, as well as clear separation among these clusters, suggesting that the different studied groups may have distinctively different metabolic profiles. These results are shown in Figs. S7A, S7C, and S8 (high-dose regimen), and Figs. S7B, S7D, and S9 (low-dose regimen). However, the lower cross-validation (CV) parameters, particularly those in the low-dose group, revealed that the generated PLS-DA models did not exhibit good discriminatory or predictive ability.

The representative LC-MS base peak chromatograms (BPCs) for all analytical assays outlined above have been shown in Figs. S10 and S11.

### 3.2 Metabolic pathway impact of IVLP

**Fig. 7:**
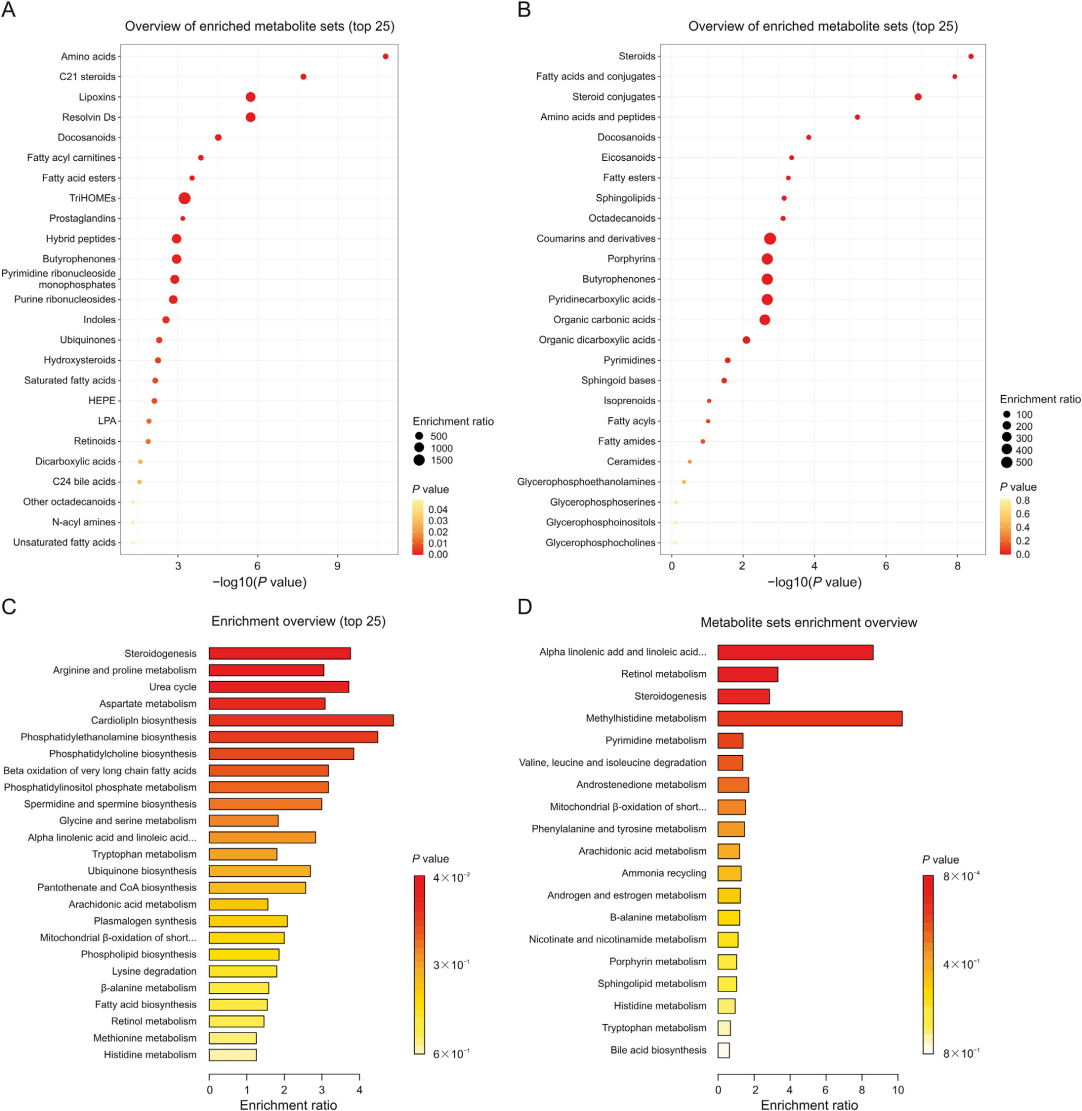
Overview of metabolite set enrichment analysis (MSEA) results for *in vivo* tissue (A, C) and perfusate (B, D) sampling. (A, B) Visualization of metabolite set enrichment based on chemical structures. Node sizes represent the total number of metabolites in each cluster set, whereas the node colour intensity indicates the higher impact of a given sub-chemical class. (C, D) The horizontal bar graphs summarize the metabolic pathways most strongly affected during lung perfusion. Pathways with a *P*-value <0.05 and a fold enrichment ≥2 were considered to have significant changes.

The significant metabolites and lipid species were then mapped onto biochemical pathways through metabolic pathways and enrichment analyses. To this end, KEGG pathway analysis was implemented in the MetaboAnalyst platform, resulting in the identification of over 20 significantly perturbed metabolic pathways in the perfusate and tissue samples (Fig. 7, and Tables S2−S4). For the tissue samples, the most impacted pathways were: (1) steroidogenesis, (2) arginine and proline metabolism, (3) the urea cycle, (4) aspartate metabolism, (5) cardiolipin biosynthesis, (6) phosphatidylethanolamine biosynthesis, (7) phosphatidylcholine biosynthesis, and (8) β-oxidation of long-chain FAs (with at least 2 altered metabolites matching to each pathway). In contrast, the analysis of the perfusate samples revealed enriched metabolite sets in the following pathways: (1) α-linolenic acid and linoleic acid metabolism, (2) retinol metabolism, (3) steroidogenesis, (4) methyl-histidine metabolism, (5) pyrimidine metabolism, (6) branched-chain AA degradation, (7) androstenedione metabolism, (8) mitochondrial β-oxidation of short-chain FAs, (9) phenylalanine and tyrosine metabolism, (10) arachidonic acid metabolism, and (11) ammonia recycling (with 2–5 compounds matching to the pathway). In addition, several less-significant (only 1 hit) pathways were also observed in the metabolomics data, with the following potentially being of particular interest: tryptophan (Trp) metabolism, porphyrin metabolism, nicotinamide metabolism, ubiquinone biosynthesis, and lysine degradation.

## 4 Discussion

While surgical resection alone is potentially curative in patients with CRC pulmonary metastases, patients frequently experience local or distant relapse [4,6]. Patients presenting with systemic disease are at the highest risk of relapse, and are generally advised to undergo systemic adjuvant chemotherapy, which has been shown to be beneficial in several cooperative group trials. However, chemotherapeutic agents can produce significant systemic toxicity and side effects. Consequently, the IVLP platform was developed to facilitate the localized delivery of high doses of anticancer drugs to the lungs during surgical resection, with the aim of preventing recurrent disease while avoiding the systemic toxicity that would otherwise occur [37]. Nonetheless, this approach remains hampered by various limitations, most of which are related to the difficulties associated with precisely monitoring drug levels and identifying determinants of the drug's cytotoxicity [22,23]. Although researchers have investigated several potential predictive factors of chemotherapy outcomes, these factors are not currently applied routinely in (pre)clinical practice [6]. Thus, an additional tool is required to prognosticate the patient's pathological response to the selected chemotherapy treatment.

Many recent studies have documented the relationship between cellular metabolism and therapeutic outcomes, including chemotherapy response and relapse [2,20]. Metabolomics—an important component of systems biology—monitors the intermediates and products of cellular metabolism, which can be affected by both xenobiotics and exogenous factors and contribute to cellular (dys)function [2]. Furthermore, the metabolome provides a phenotypic evaluation of cellular and systemic health, with potential implications for disease pathogenesis studies, biomarker discovery, drug effectiveness evaluation, prognosis prediction, and personalized medicine. In this context, spatial metabolomics is of the utmost importance, as it enables the direct visualization of the distribution of metabolites in tissues, thus providing an in-depth understanding of disease- or treatment-associated biochemical changes within specific structures [14]. In the present study, we characterized metabolic alterations caused by the delivery of high-doses of OxPt to the lung via IVLP by deploying untargeted metabolomics profiling alongside spatial SPME microprobing to compare metabolite patterns in tissue and perfusate samples obtained at baseline and after a 3h-drug treatment. Integrated screening based on univariate and multivariate analysis revealed 193 altered features in tissue extracts and 327 dysregulated variables in perfusate, of which a quarter were successfully identified by matching them to library spectra or through computational structural dereplication [34,36]. The altered metabolites and lipids were enriched in pathways involving central carbon metabolism, FA oxidation, α-linolenic and linoleic acid metabolism, arachidonic acid metabolism, and steroidogenesis.

Specifically, the enrichment analysis revealed that arachidonic acid metabolism, α-linolenic acid metabolism, and linoleic acid metabolism were the most significantly enriched pathways that could be linked with a massive release of lipid mediators responsible for controlling the inflammatory response in the perfused lung. Indeed, acute inflammatory response, which was presumably initiated by the lung injury, was recognized as a distinct feature of the dysregulated metabolipidome. This was particularly manifested by the strong release of PUFA arachidonic-acid-derived prostaglandins and leukotrienes (i.e., prostaglandin E1; leukotriene C4, D4, E4; 10,11-dihydro-12-hydroxy-leukotriene E4; 12-oxo-20-trihydroxy-leukotriene B4). Although acute inflammation is an innate immune defence that protects the host from systemic infection, thus helping to restore tissue homeostasis, uncontrolled inflammatory response (in terms of magnitude or duration) can cause apparent lung injury and potentially pulmonary fibrosis, which severely impairs essential gas-exchange processes [38,39]. Unresolved lung inflammation or acute lung injury can progress to chronic inflammation, which occurs in common lung diseases such as chronic obstructive pulmonary disease, acute respiratory distress syndrome, and asthma. Furthermore, successfully alleviating inflammation depends on the phagocytic clearance of apoptotic granulocytes and achieving a balance between different sets of mediators and specific receptors, which ultimately leads to the phenotype switching from a pro-inflammatory cell to a more anti-inflammatory/pro-resolution phenotype [39]. Our findings indicated that the release of lipoxins diminished throughout 3h-period of the lung perfusion, thus the production of pro-inflammatory leukotrienes was not ceased. Adding to the above, the release of pro-resolving bioactive lipids increased only moderately (e.g. resolvins (RvDs)). These findings may indicate unsuccessful resolution triggering and sustaining pulmonary chronic inflammation.

We also found that lung injury promoted alterations in energy metabolism and mitochondrial dysfunction. The lung is frequently overlooked as a metabolically active organ, but biochemical studies have clearly demonstrated that its glucose utilization surpasses that of many other organs, including the brain, heart, and kidney [40]. Furthermore, the lung possesses the metabolic flexibility to adapt to changes in energy demand. In addition, whereas some tissues preferentially utilize FAs for much of their energy production (e.g., heart), prior findings suggest the lung primarily utilizes this energetic pathway during times of nutrient deprivation [41,42]. Indeed, the results of this study support these previous findings indicating the accelerated consumption of FAs as an emergency fuel under conditions of glucose deprivation. However, inefficient medium- and long-chain FA combustion in the muscle was also observed, as evidenced by the accumulation of medium- and long-chain acylcarnitines (e.g., octanoylcarnitine, undecanoylcarnitine, 2-hydroxylauroylcarnitine, 2-dodecenoylcarnitine, tetradecanoylcarnitine hexadecenoylcarnitine). It is well-documented that when provision of acyl-CoAs exceeds the capacity for FA β-oxidation, acyl-CoAs may accumulate inside the mitochondria and drive the carnitine palmitoyltransferase-2 (CPT2) reaction in reverse, producing acylcarnitines, which are subsequently secreted [40]. This phenomenon may have also been present in our study accompanied by diminished availability of short-chain acylcarnitines. This possibility is further supported by the increased levels of N-acylglycine species (palmitoylglycine, octanoylglycine and oleoylglycine) observed during IVLP, as the accumulation of these species is common in disorders of mitochondrial FA β-oxidation [40]. Furthermore, when β-oxidation is blocked, the ω-oxidation of specific FAs can be upregulated to limit their accumulation (or the accumulation of their intermediates) and, consequently, the detrimental effects of the discussed disorders [43]. The findings of this research appear to corroborate this theory, as increased production of hydroxylated FAs and dicarboxylic FAs was observed (i.e., hydroxyoctanoic acid, decanedioic acid, dodecanedioic acid, tetradecanedioic acid). Notably, the breakdown of FAs can place significant oxidative stress on cells, particularly those not adequately equipped to handle an acute rise in the generation of reactive oxygen species (ROS) [40,44]. Although ROS are essential for physiologic functions (i.e., by acting as a second messenger), oxidative/antioxidative imbalance can be detrimental, as it promotes mitochondrial damage. Indeed, we identified several markers of oxidative activity (e.g., cyclic 6-hydroxymelatonin, carnosine, ubiquinone-1), along with those involved in mitochondrial membrane remodelling (lysophosphatidylethanolamine (p-16:0), phosphatidylserine (38:0)). Both of the above markers are indicative of oxidative damage and dysfunction in the mitochondrial dynamics, which both alter cell bioenergetics and hinder lung recovery after an injury [44]. Accordingly, the abnormality of the mitochondrial structure coupled with energy metabolism disorders likely produces a vicious cycle wherein ATP production is limited and the production of reactive species increases.

Altered AA metabolism also played a major role in the group classification of IVLP samples. Specifically, an increase in branched-chain AAs was recognized as a likely indicator of the muscle breakdown expected in cases of lung injury [45]. Adding to the above, the metabolic pathway enrichment analysis revealed that the metabolism of phenylalanine and tryptophan, together with their derivatives, were the most significantly enriched pathways among AAs. Prior studies have found that phenylalanine hydroxylase (PAH) activity responsible for converting phenylalanine to tyrosine tends to be dysfunctional in inflammatory-related disease states [46,47]. This raises the possibility that the circulating phenylalanine levels observed in our study may have been altered due to the reduced action of the PAH enzyme. Trp catabolism is another important factor in the lung microenvironment, as it directly influences immune responses [48]. Trp catabolism occurs predominantly through the activation of the enzyme, indoleamine 2,3-dioxygenase (IDO), which in turn produces the metabolites of the kynurenine (Kyn) pathway. Surprisingly, we found that Trp levels were reduced only in the early stages of IVLP. This was consistent with increased catabolism via the IDO pathway, as demonstrated by an increase in Kyn/Trp. Since most of the effects of Trp catabolism emanate from the accumulation of its active metabolites (e.g., kynurenine) rather than Trp depletion [48], the accelerated generation of kynurenine through IDO activation (observed in our study) may potentially induce immunologic tolerance and anti-inflammatory effects. Interestingly, the level of aminoadipic acid—an intermediate in the breakdown of lysine that has been shown to inhibit the production of kynurenic acid [49]—remained unaltered during IVLP, but significantly increased during blood reperfusion. This finding further suggests that kynurenine production is stimulated during the chemo-perfusion of the lung.

Finally, steroidogenesis, specifically aldosterone synthesis, was largely upregulated during IVLP. In the lung, the chronic activation of the renin-angiotensin-aldosterone system (RAAS), which regulates cell proliferation, immune-inflammatory response, hypoxia, and angiogenesis, contributes to lung injury and different pulmonary diseases [50,51]. Additionally, angiotensin metabolites and aldosterone may activate oxidant-stress-signalling pathways, which decrease levels of bioavailable nitric oxide, increase inflammation, and promote the spread of cells, extracellular matrix remodelling, and, ultimately, the development of fibrosis. Moreover, persistently elevated levels of aldosterone can potentially activate the mineralocorticoid receptors in vascular and muscle cells to initiate the signalling pathways that promote endothelial dysfunction, vascular remodelling, and, consequently, impaired vascular reactivity [51].

This study makes a number of key contributions. As far as we know, the literature does not contain any metabolomics studies exploring changes in metabolite patterns or levels during the local delivery of OxPt to the lung. In particular, the discovery of biomarkers in a non-invasive and convenient manner has remained an especially pressing challenge in recent years. Using a spatial SPME-based microprobe in conjunction with global metabolic profiling-based screening, enabled us to successfully identify distinct chemotherapy-related metabolic alterations. Although this is not the first time when low invasive *in vivo* tissue sampling has been used during localized delivery of high-dose chemotherapy to the lung [25,52], there are many advantages of the current study that need to be highlighted. Precisely, while the previous studies involving the local administration of doxorubicin to the porcine/human lung or FOLFOX (being a combination of therapies involving folinic acid, 5-fluorouracil and oxaliplatin) to the porcine lung were rather proof-of-concept studies with a limited number of cases enrolled; the current work presents results from the complete pre-clinical trial that permits the formulation of solid biological conclusions in relation to other biochemical indices or pulmonary hemodynamic parameters. Adding to the above, as this was a porcine IVLP 3-day survival study, we paid particular attention to the experimental protocol which was designed to ensure minimal tissue injury during sampling. Accordingly, the sharpened fibers of 280 μm in diameter with an 8-mm long coated section were employed for analytes' extraction; while in previous porcine studies, fibers with a blunt tip were utilized which were also more densely distributed in the relevant sections of the sampled lung. Finally, the proposed protocol involving two variants of microprobes coated with C18-bonded porous silica sorbent and mixed-mode phase enabled a wide range of analytes profiling that was not feasible in previous studies when only one type of coating was employed. Collectively, several improvements in the study protocol next to a comprehensive analysis of various matrixes (lung tissue, perfusate, plasma) permitted deciphering the metabolic landscape during the local administration of OxPt via IVLP. Despite this success, it is important to acknowledge some of this study's limitations. First, a larger sample size and an independent validation cohort will be necessary to confirm the validity of our metabolic biomarkers of lung injury. Second, only a fraction of the altered metabolites and lipids were successfully identified, which was attributed to the difficulty in identifying variables generally encountered in untargeted profiling approaches. Finally, while we examined metabolic alterations in response to OxPt in normal tissue and cells, we did not explore it in the target cancer cells. Nonetheless, despite the above limitations, we ultimately achieved our objective: to identify potential metabolic biomarkers of ALI using a novel sensitive analytical methodology capable of detecting a wide variety of endogenous and exogenous compounds.

## 5 Conclusions

The present study has shown that spatiotemporally resolved pharmacometabolomics can be combined with chemometric tools to detect consistent metabolic alterations and predict the pathological response to high-dose adjuvant chemotherapy. The metabolic patterns in the lung tissue and perfusate samples changed dramatically over the course of lung chemo-perfusion. A distinct panel of metabolic biomarkers was identified to highlight relevant mechanistic explorations of the lung injury. The altered metabolites and lipid species were particularly enriched in pathways involving the metabolism of arachidonic acid, AAs, FAs, and aldosterone synthesis. Together, the findings presented herein provide a paradigm for the investigation of global changes in metabolism throughout IVLP in order to improve ALI diagnosis and make adjustments to the utilized chemotherapy regimen.

## CRediT author statement

**Mariola Olkowicz:** Conceptualization, Methodology, Validation, Formal analysis, Investigation, Data curation, Writing - Original draft preparation, and Reviewing and Editing, Visualization; **Khaled Ramadan:** Conceptualization, Methodology, Validation, Formal analysis, Investigation, Data curation, Writing - Reviewing and Editing; **Hernando Rosales-Solano:** Methodology, Formal analysis, Investigation, Writing - Reviewing and Editing; **Miao Yu:** Software, Validation, Formal analysis, Investigation, Data curation, Writing - Reviewing and Editing; **Aizhou Wang:** Methodology, Investigation, Writing - Reviewing and Editing; **Marcelo Cypel:** Conceptualization, Supervision, Funding acquisition, Resources, Project administration, Writing - Reviewing and Editing; **Janusz Pawliszyn:** Conceptualization, Supervision, Funding acquisition, Resources, Project administration, Writing - Reviewing and Editing.

## Declaration of competing interest

The authors declare that there are no conflicts of interest.
