# Supplementary material for: Mapping the metabolic responses to oxaliplatin-based chemotherapy with in vivo spatiotemporal metabolomics
Source: J Pharm Anal. 2023 Aug 9;14(2):196–210. doi: 10.1016/j.jpha.2023.08.001 (PMC10921245; doi:10.1016/j.jpha.2023.08.001)
Supplement: Multimedia component 1 [file mmc1.docx]

**SUPPLEMENTARY FILE**

**Mapping the metabolic responses to oxaliplatin-based chemotherapy with in vivo spatiotemporal metabolomics**

Mariola Olkowicz ^a, b, 1 **^, Khaled Ramadan ^c, 1^, Hernando Rosales-Solano ^a^, Miao Yu ^d^, Aizhou Wang ^c^ , Marcelo Cypel ^c, e^, Janusz Pawliszyn ^a *^

^a^ Department of Chemistry, University of Waterloo, Waterloo, ON, Canada;

^b^ Jagiellonian Centre for Experimental Therapeutics (JCET), Jagiellonian University, Krakow, Poland;

^c^ Latner Thoracic Surgery Research Laboratories, Toronto General Hospital Research Institute, University Health Network, Toronto, ON, Canada;

^d^ The Jackson Laboratory, JAX Genomic Medicine, Farmington, CT, USA;

^e^ Division of Thoracic Surgery, Department of Surgery, University Health Network, University of Toronto, Toronto Lung Transplant Program, Toronto, ON, Canada

^1^ These authors have contributed equally to this work;

* Corresponding author: Department of Chemistry, University of Waterloo, Waterloo, ON, Canada; ** Corresponding author: Jagiellonian Centre for Experimental Therapeutics (JCET), Jagiellonian University, Krakow, Poland;

*E-mail addresses:* janusz@uwaterloo.ca (J. Pawliszyn); mariola.olkowicz@uj.edu.pl (M. Olkowicz).

Supplementary methods

**Table S1.** *LC method details and MS acquisition parameters for the analysis of metabolites and lipid species in SPME-based extracts.*

| Ultra-High-Performance Liquid Chromatography | | |
| --- | --- | --- |
| Metabolomic Investigations Lipidomic Investigations | | |
| Column | Supelco Discovery HS F5, 120 Å, 3 μm, 100 mm × 2.1 mm | Waters XSelect CSH C18, 130Å, 3.5 µm, 75 mm × 2.1 mm |
| Mobile phase  Phase A    Phase B | H_2_O + 0.1% FA (+ 1 mM AA)  ACN + 0.1% FA (+ 1 mM AA) | MeOH/H_2_O 40:60 + 10 mM AcNH_4_ + 1 mM AA (+ 0.02% AA)  IPOH/MeOH 90:10 + 10 mM AcNH_4_ + 1 mM AA (+ 0.02% AA) |
| Gradient programme | 0–3 min 0% B; 3–25 min 0–90% B; 25–34 min 90% B; 34–35 min 90–0% B; 35–40 min 0% B | 0–2 min 20% B; 2–3 min 20–30% B; 3–13.5 min 30–80% B; 13.5–18.5 min 80–85% B; 18.5–20 min 85–95% B; 20–21.5 min 95% B; 21.5-27 min 20% B |
| Flow rate [µL/min] | 300 | 300 |
| Column temperature [°C] | 25 | 55 |
| Autosampler temperature [°C] | 4 | 4 |
| Injection volume [µL] | 10 | 10 |
| High-Resolution, Accurate-Mass Mass Spectrometry (HESI Ion Source) | | |
| Acquisition mode Full Scan | | |
| Mass range [m/z] 100 – 1000 | | |
| Max. injection time [ms] 100 | | |
| Automatic gain control Balanced: 1e6 | | |
| Resolution High: 50,000 at 2 Hz (FWHM* 200 m/z) | | |
| Electrospray voltage [kV] | 4.0 (-2.9) | 3.5 (-2.9) |
| Sheath gas | 55 | 30 |
| Auxiliary gas | 30 | 10 |
| Sweep gas | 5 | 2 |
| Capillary temperature [°C] | 300 (300) | 300 (300) |
| Vaporizer temperature [°C] | 300 | 300 |
| Lock mass m/z 391.2843 (ve+); m/z 255.2329 (ve−) | | |

FA – formic acid; AA – acetic acid; AcNH_4_ – ammonium acetate; ESI– mode parameters in brackets; *Full-width at half maximum (FWHM).

Supplementary results

**A Lung/ESI+ B**


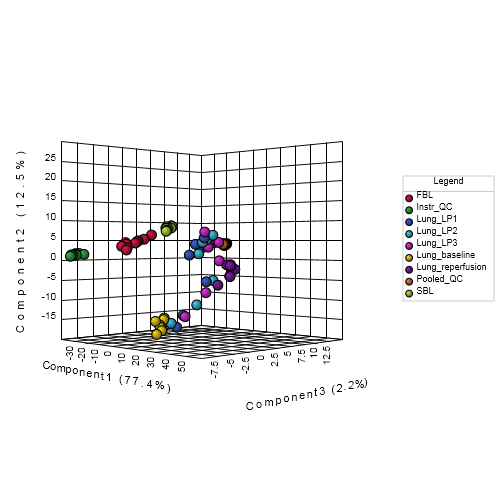

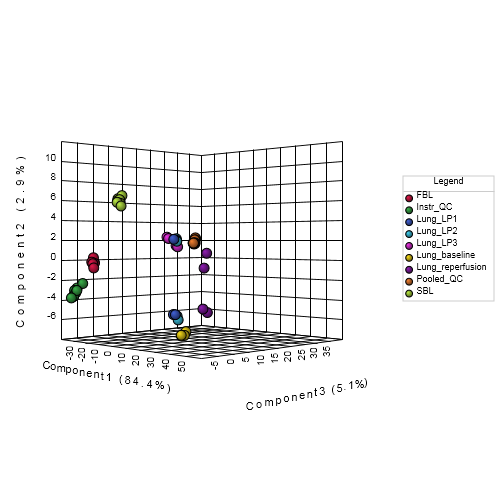


**C Lung/ESI- D**


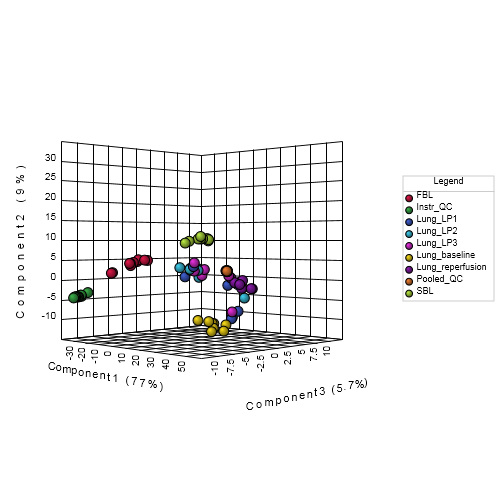

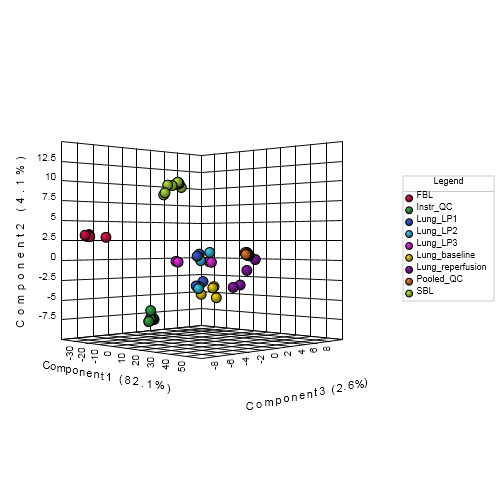


**Fig. S1.** *3D Partial least squares-discriminant analysis (PLS-DA) score plots for data obtained via (****A****,* ***B***) *SPME–RP/LC–MS ESI (+) and (****C****,* ***D****) SPME–RP/LC–MS ESI (–) from lung sampling during (****A****,* ***C****) high-dose and (****B****,* ***D****) low-dose regimens*. Abbreviations: **FBL/SBL**: fiber/solvent blank samples; **Instr_QC**: instrumental quality control samples; **Pooled_QC**: pooled quality control samples; **Lung_baseline**: samples collected pre-perfusion; **Lung_LP1/LP2/LP3**: samples collected at the 1^st^, 2^nd^, and 3^rd^ hour of in vivo lung perfusion (IVLP); **Lung_reperfusion**: samples collected 30 min post reperfusion; **ESI+**: positive ionization mode; **ESI–**: negative ionization mode.

**A SN/MM/ESI+ B**


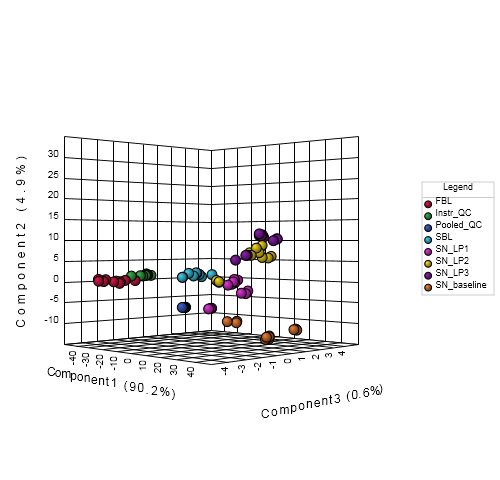

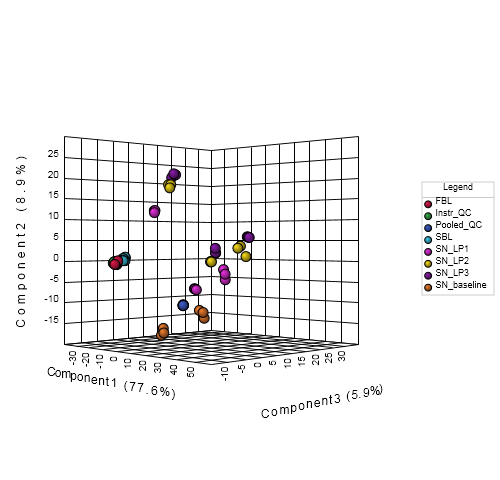


**C SN/MM/ESI- D**


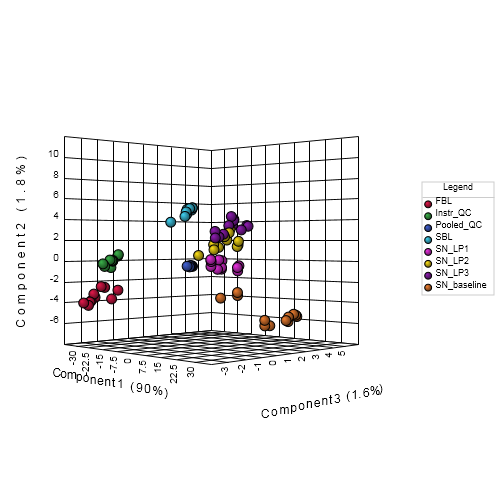

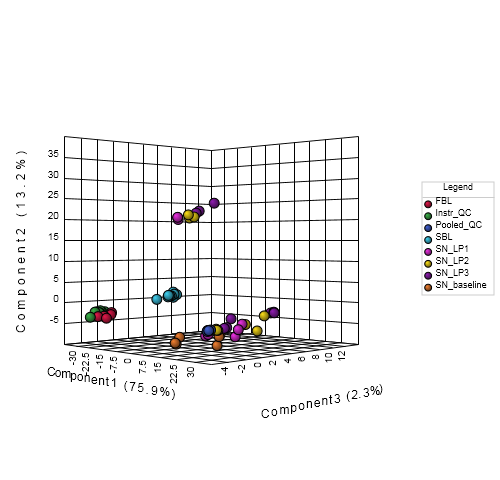


**Fig. S2.** *3D Partial least squares-discriminant analysis (PLS-DA) score plots for data obtained via (****A****,* ***B****) SPME–RP/LC–MS ESI (+) and (****C****,* ***D****) SPME–RP/LC–MS ESI (–) from perfusate sampling during (****A****,* ***C****) high-dose and (****B****,* ***D****) low-dose regimens*. Abbreviations: **SN**: supernatant/perfusate samples; **Instr_QC**: instrumental quality control samples; **Pooled_QC**: pooled quality control samples; **FBL/SBL**: fiber/solvent blank samples; **SN_baseline**: samples collected before oxaliplatin (OxPt) administration; **SN_LP1/LP2/LP3**: samples collected at the 1^st^, 2^nd^, and 3^rd^ hour of in vivo lung perfusion (IVLP); **MM**: mixed-mode-based SPME coating; **ESI+**: positive ionization mode; **ESI–**: negative ionization mode.

**A SN/C18/ESI+ B**


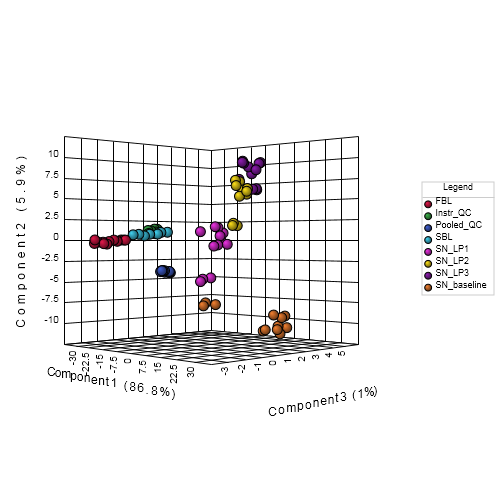

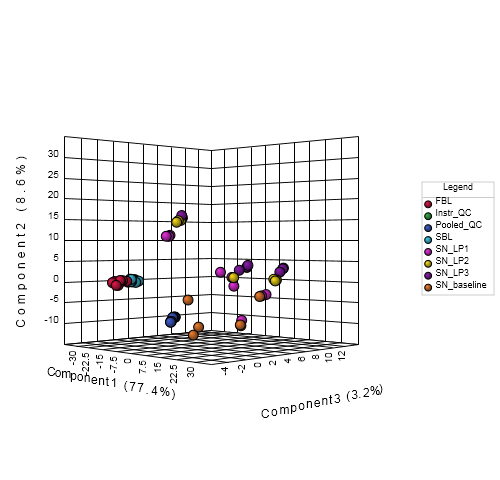


**C SN/C18/ESI- D**


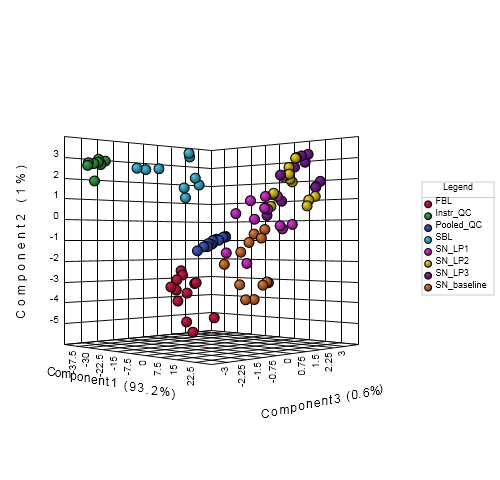

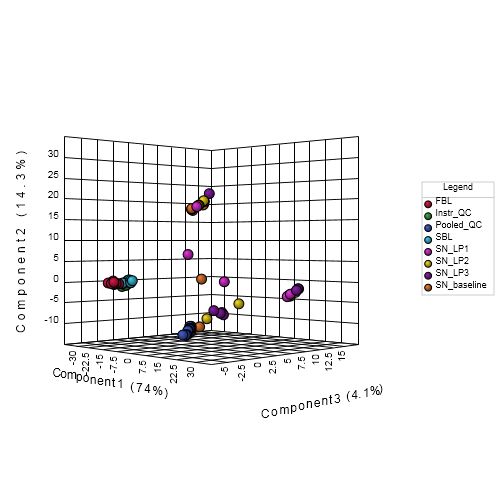


**Fig. S3.** *3D Partial least squares-discriminant analysis (PLS-DA) score plots for data obtained via (****A****,* ***B****) SPME–RP/LC–MS ESI (+) and (****C****,* ***D****) SPME–RP/LC–MS ESI (–) from perfusate sampling during (****A****,* ***C****) high-dose and (****B****,* ***D****) low-dose regimens*. Abbreviations: **SN**: supernatant/perfusate samples; **Instr_QC**: instrumental quality control samples; **Pooled_QC**: pooled quality control samples; **FBL/SBL**: fiber/solvent blank samples; **SN_baseline**: samples collected before oxaliplatin (OxPt) administration; **SN_LP1/LP2/LP3**: samples collected at the 1^st^, 2^nd^, and 3^rd^ hour of in vivo lung perfusion (IVLP); **C18**: C18-based SPME coating; **ESI+**: positive ionization mode; **ESI–**: negative ionization mode.

**A Lung/ESI+ B**


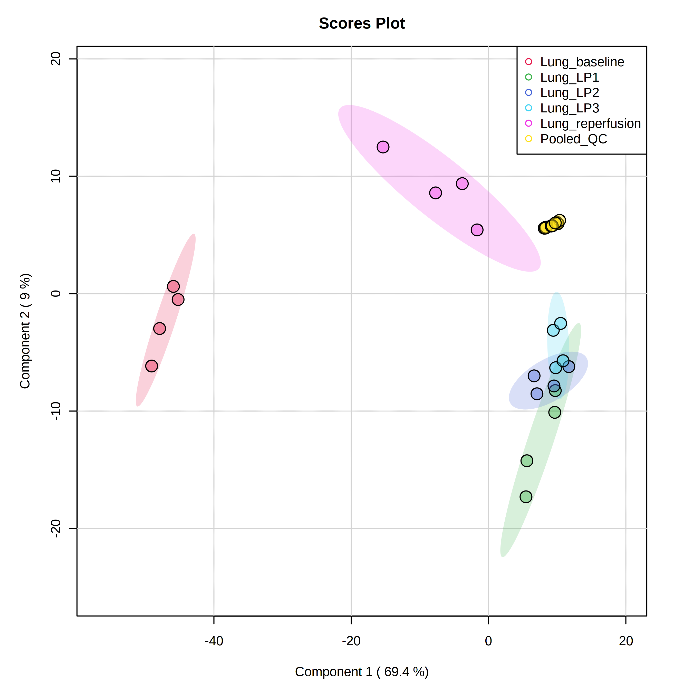

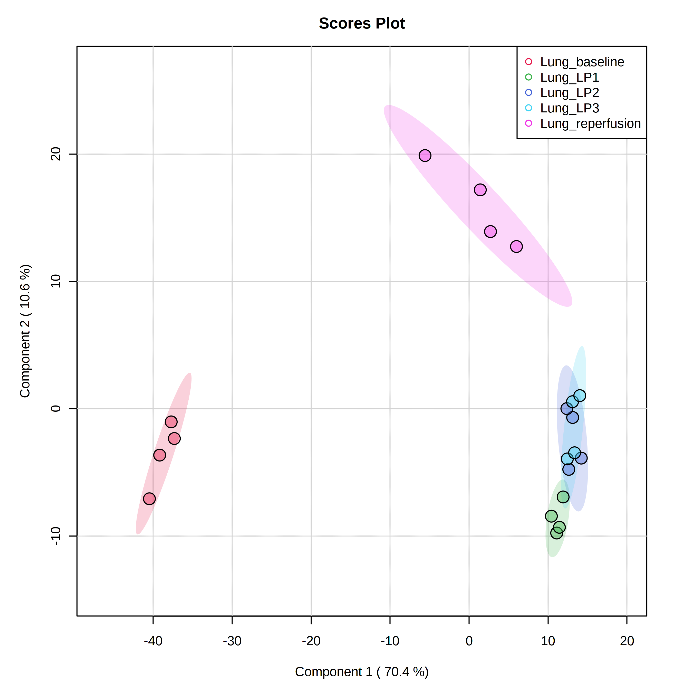


**C Lung/ESI- D**


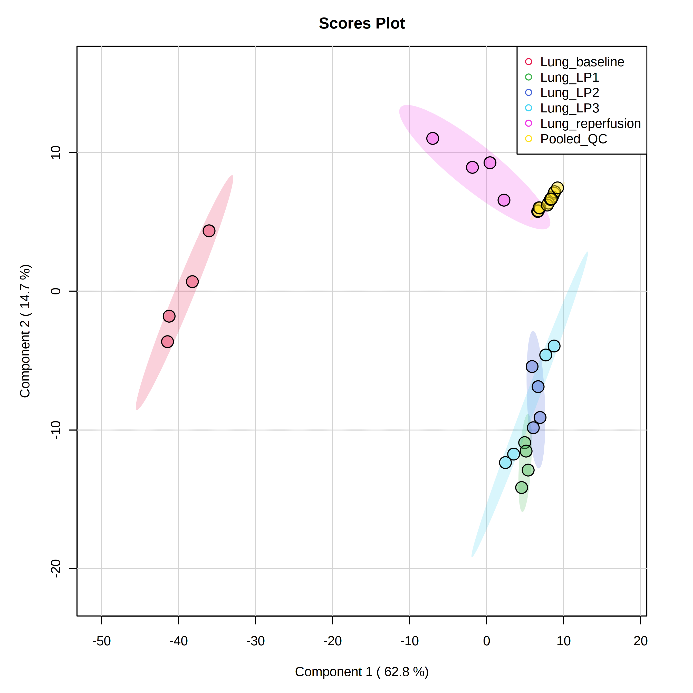

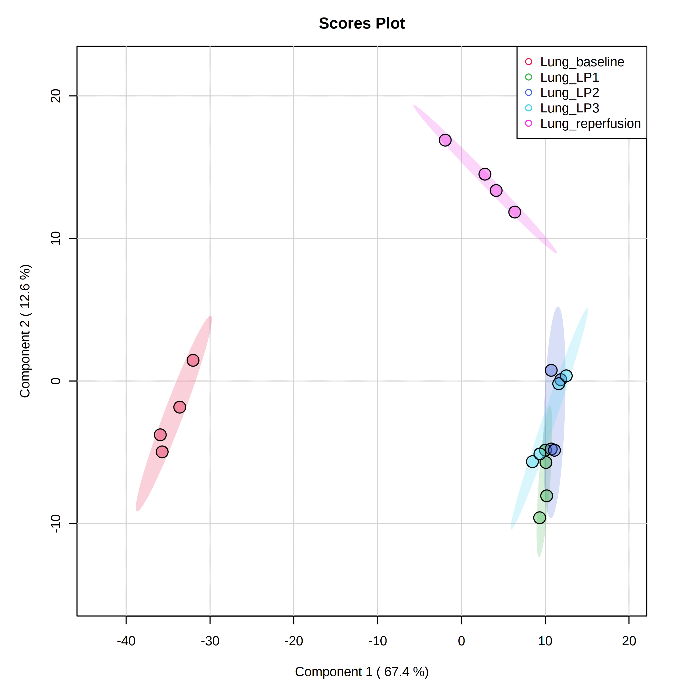


**Fig. S4.** *Partial least squares-discriminant analysis (PLS-DA) based on metabolites determined in lung tissue via SPME–LC/MS during the application of a low-dose regimen*. Abbreviations: **Lung_baseline**: samples collected pre-perfusion; **Lung_LP1/LP2/LP3**: samples collected at the 1^st^, 2^nd^, and 3^rd^ hour of in vivo lung perfusion (IVLP); **Lung_reperfusion**: samples collected 30 min post reperfusion; **Pooled_QC**: pooled quality control samples.

**A SN/MM/ESI+ B**


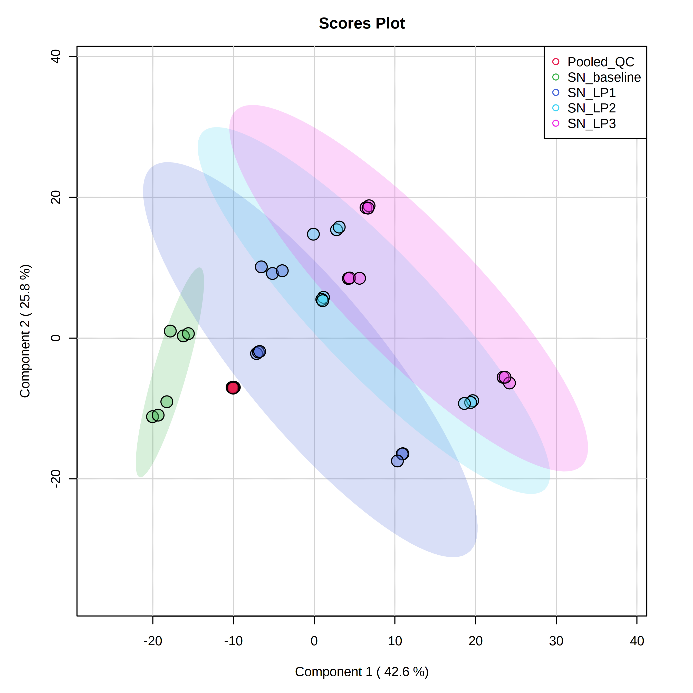

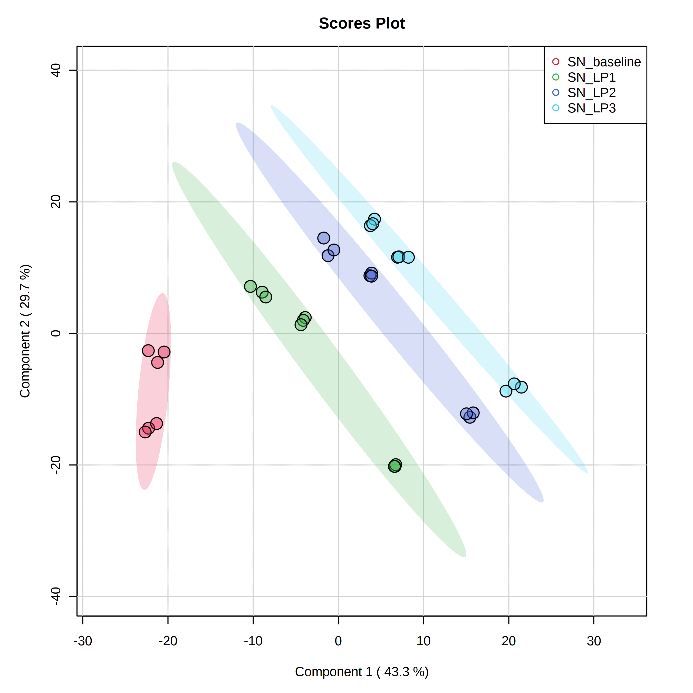


**C SN/MM/ESI- D**


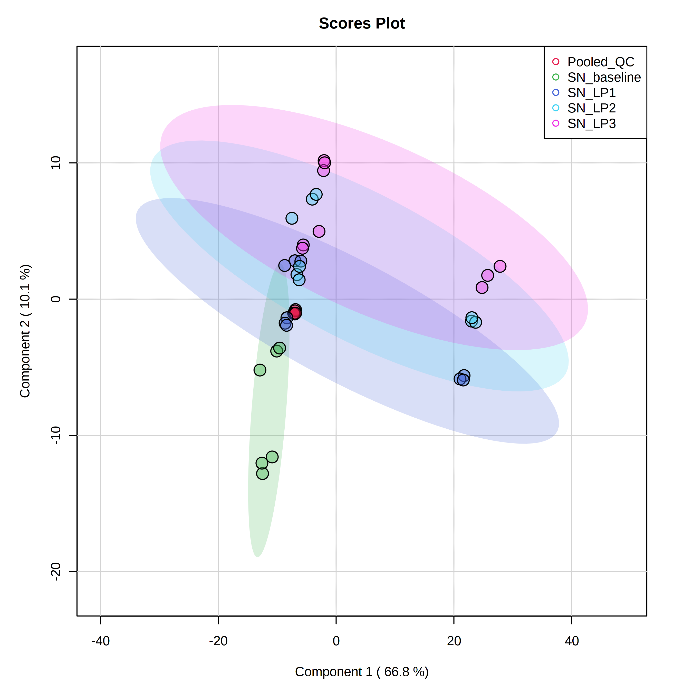

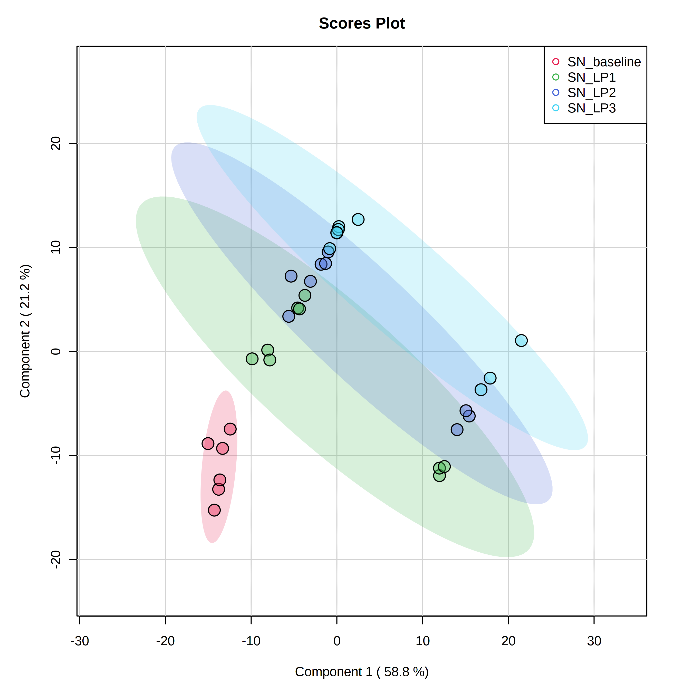


**Fig. S5.** *Partial least squares-discriminant analysis (PLS-DA) based on metabolites determined in perfusate via SPME(MM)–LC/MS during the application of a low-dose regimen*. Abbreviations: **SN**: supernatant (perfusate) samples; **Pooled_QC**: pooled quality control samples; **SN_baseline**: samples collected before oxaliplatin (OxPt) administration; **SN_LP1/LP2/LP3**: samples collected at the 1^st^, 2^nd^, and 3^rd^ hour of in vivo lung perfusion (IVLP); **MM**: mixed-mode-based SPME coating; **ESI+**: positive ionization mode; **ESI–**: negative ionization mode.

**A SN/C18/ESI+ B**


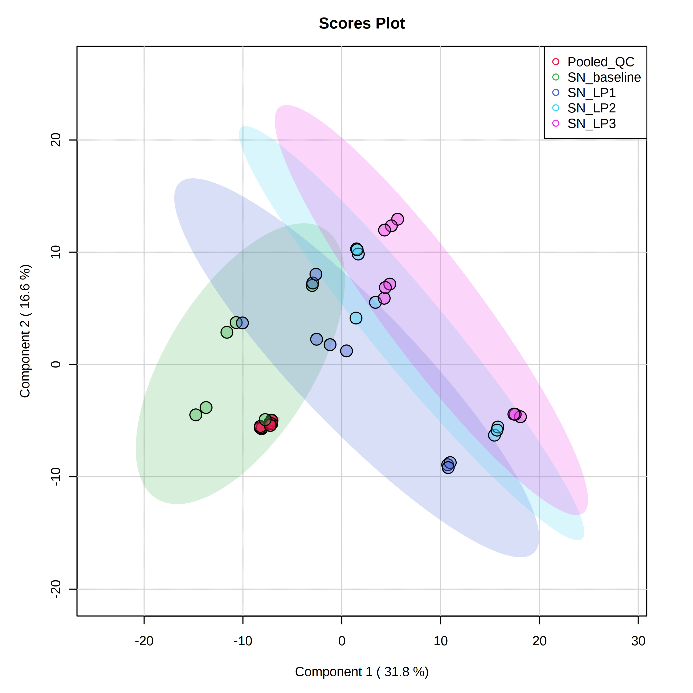

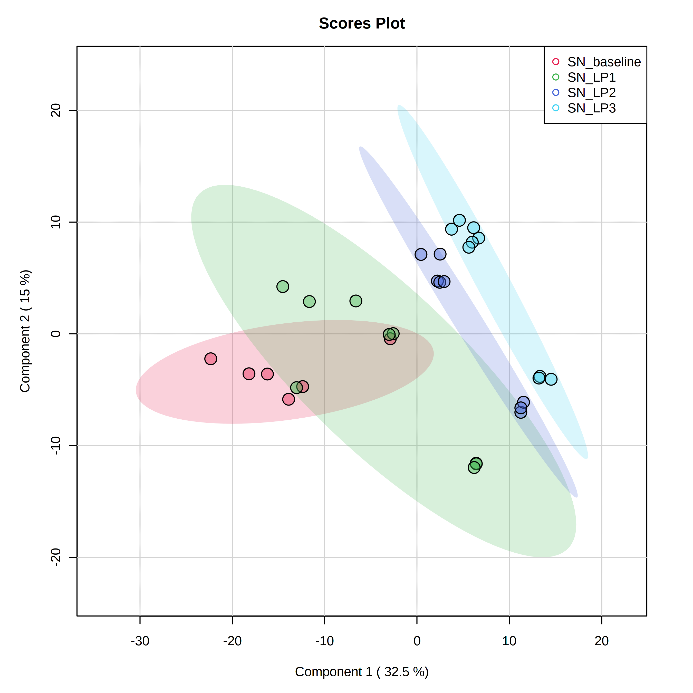


**C SN/C18/ESI- D**


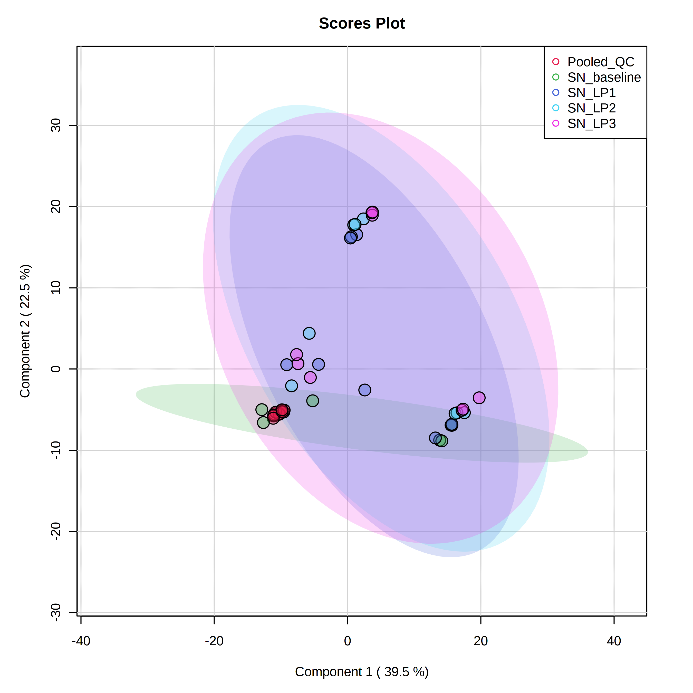

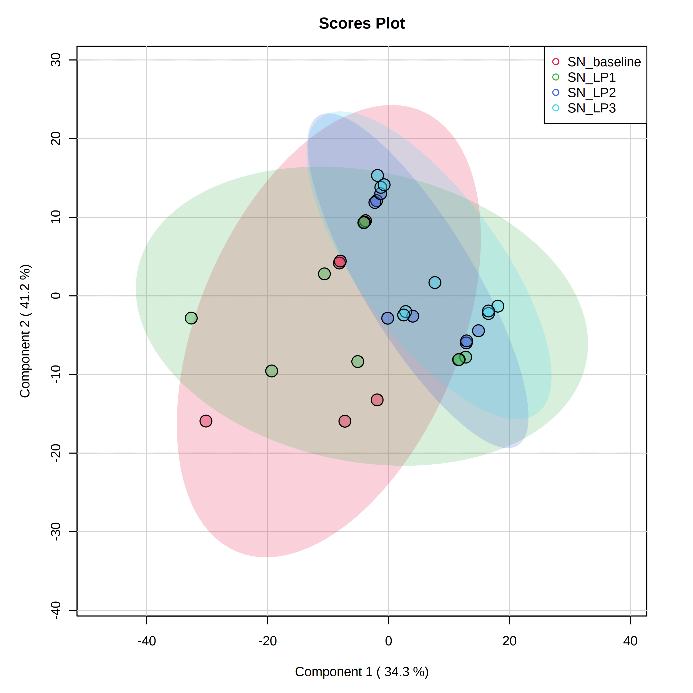


**Fig. S6.** *Partial least squares-discriminant analysis (PLS-DA) based on lipids determined in perfusate via SPME(C18)–LC/MS during the application of a low-dose regimen*. Abbreviations: **SN**: supernatant (perfusate) samples; **Pooled_QC**: pooled quality control samples; **SN_baseline**: samples collected before oxaliplatin (OxPt) administration; **SN_LP1/LP2/LP3**: samples collected at the 1^st^, 2^nd^, and 3^rd^ hour of in vivo lung perfusion (IVLP); **C18**: C18-based SPME coating; **ESI+**: positive ionization mode; **ESI–**: negative ionization mode.

**A PL/MM/ESI+ B**


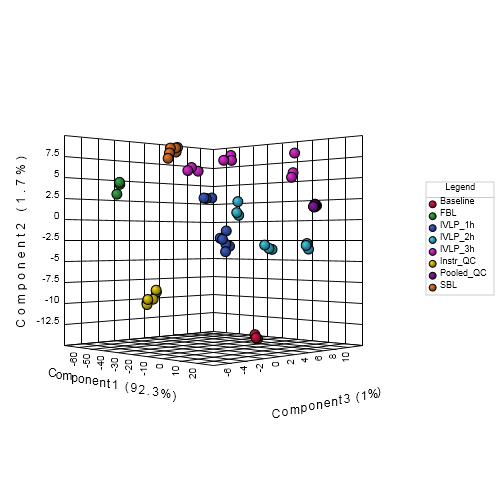

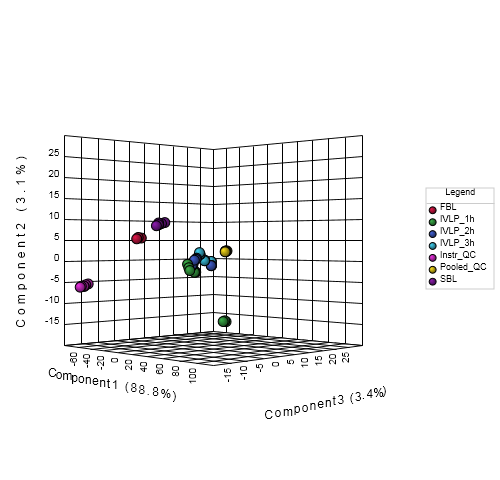


**C PL/MM/ESI- D**


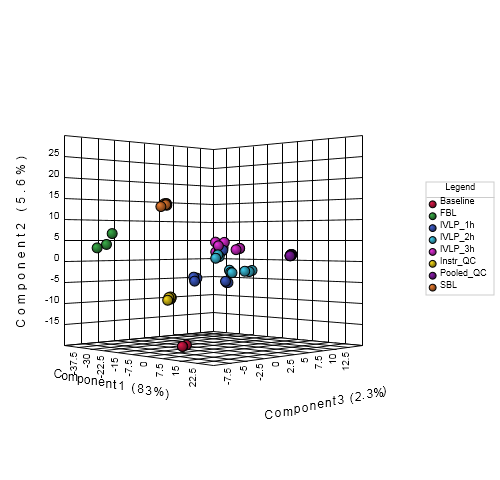

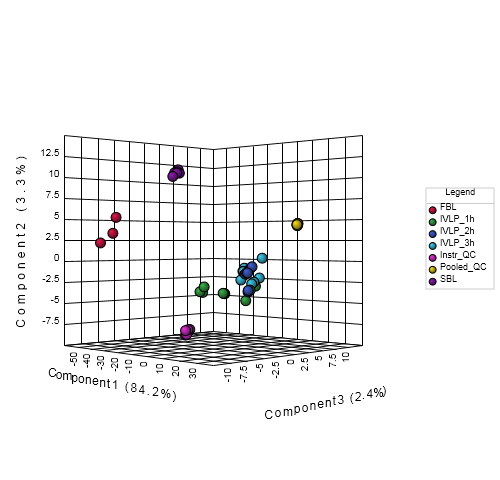


**Fig. S7.** *3D* *Partial least squares-discriminant analysis (PLS-DA) score plots for data obtained via (****A****,* ***B****) SPME–RP/LC–MS ESI (+) and (****C****,* ***D****) SPME–RP/LC–MS ESI (–) from plasma in porcine models when applying (****A****,* ***C****) high-dose and (****B****,* ***D****) low-dose regimens*. Abbreviations: **FBL/SBL**: fiber/solvent blank samples; **Instr_QC**: instrumental quality control samples; **Pooled_QC**: pooled quality control samples; **Baseline**: samples collected before oxaliplatin (OxPt) administration; **IVLP_1h/2h/3h**: samples collected at the 1^st^, 2^nd^, and 3^rd^ hour of in vivo lung perfusion (IVLP); **PL**: plasma samples; **MM**: mixed-mode-based SPME coating; **ESI+**: positive ionization mode; **ESI–**: negative ionization mode.

**A PL/MM/ESI+ B**


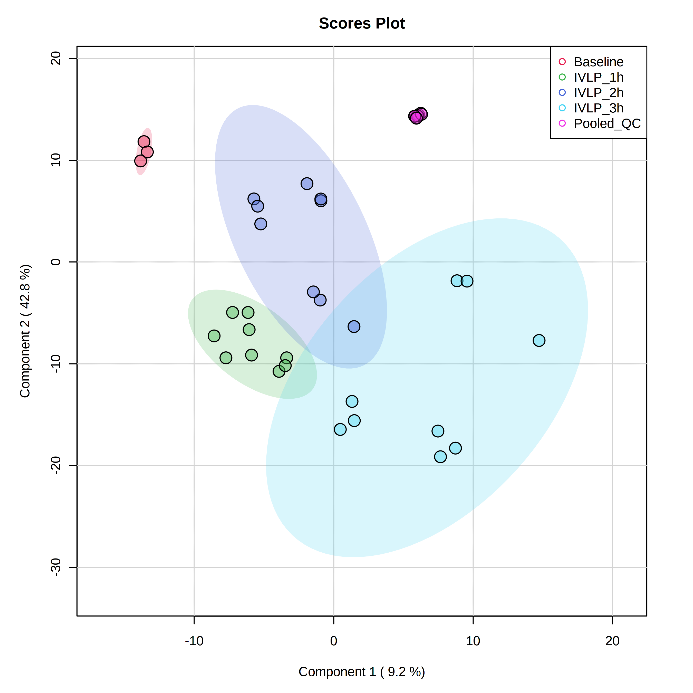

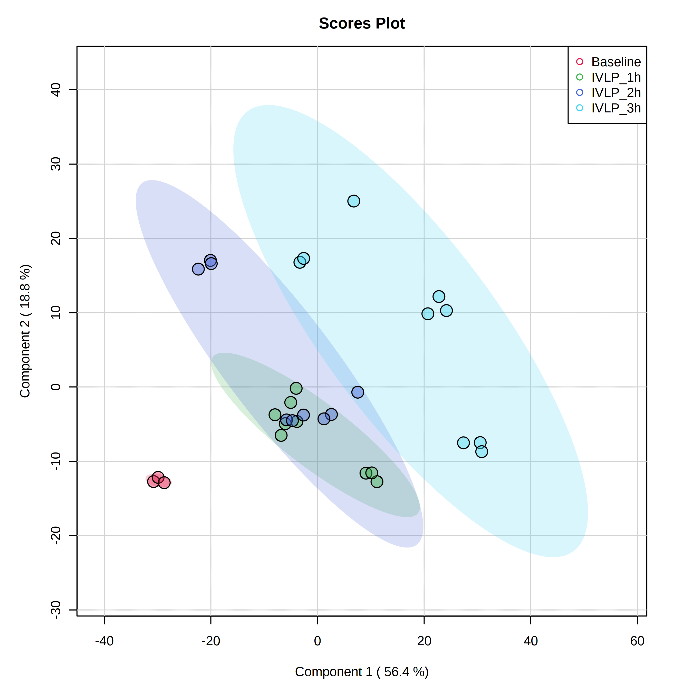


**C PL/MM/ESI- D**


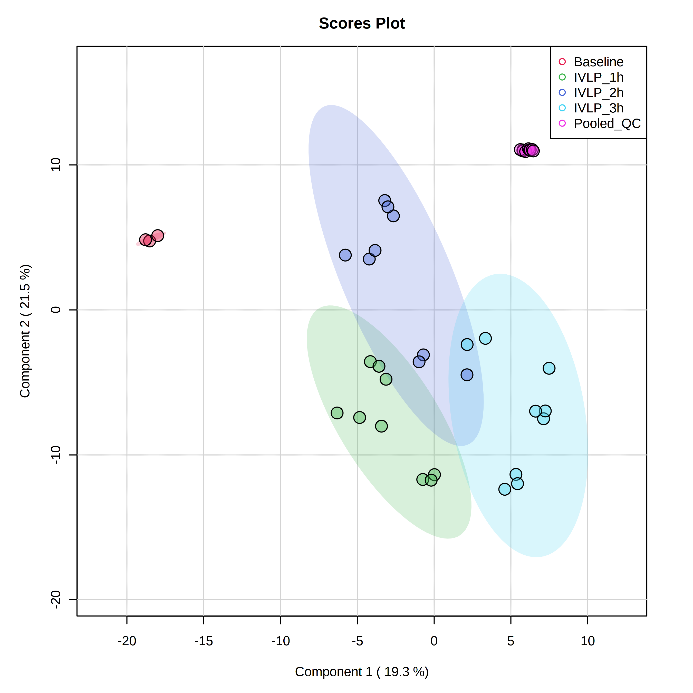

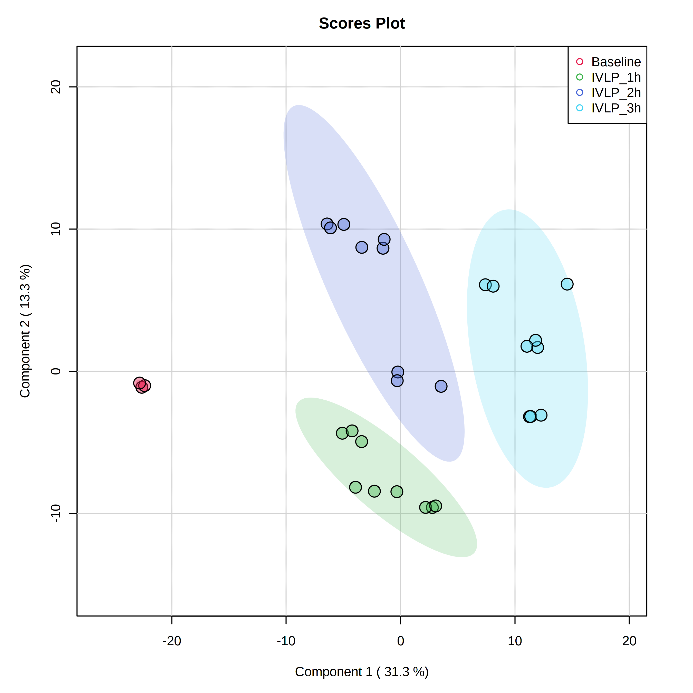


**Fig. S8.** *2D* *Partial least squares-discriminant analysis (PLS-DA) score plots for data obtained via SPME–RP/LC–MS ESI (+) (****A****,* ***B****) and SPME–RP/LC–MS ESI (–) (****C****,* ***D****) from plasma of porcine models using high-dose regimen*. Abbreviations: **Pooled_QC**: pooled quality control samples; **Baseline**: samples collected before oxaliplatin (OxPt) administration; **IVLP_1h/2h/3h**: samples collected at the 1^st^, 2^nd^, and 3^rd^ hour of in vivo lung perfusion (IVLP); **PL**: plasma samples; **MM**: mixed-mode-based SPME coating; **ESI+**: positive ionization mode; **ESI–**: negative ionization mode.

**A PL/MM/ESI+ B**


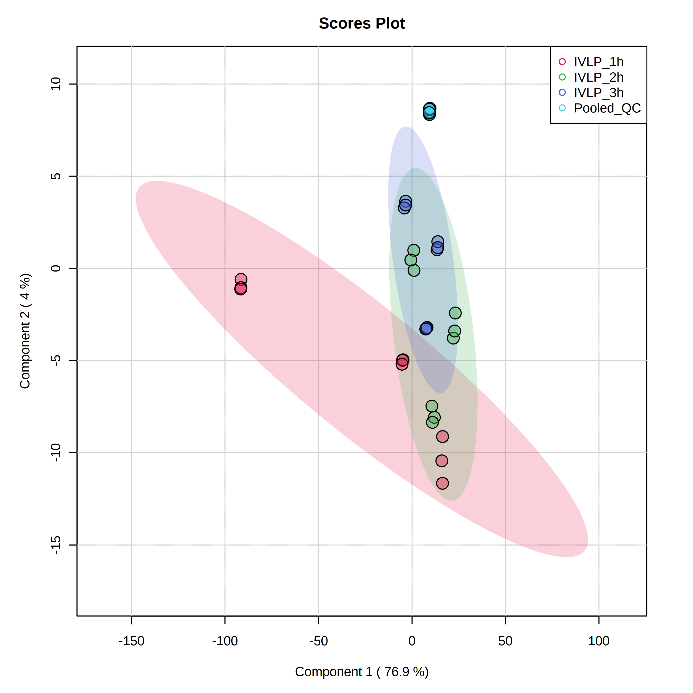

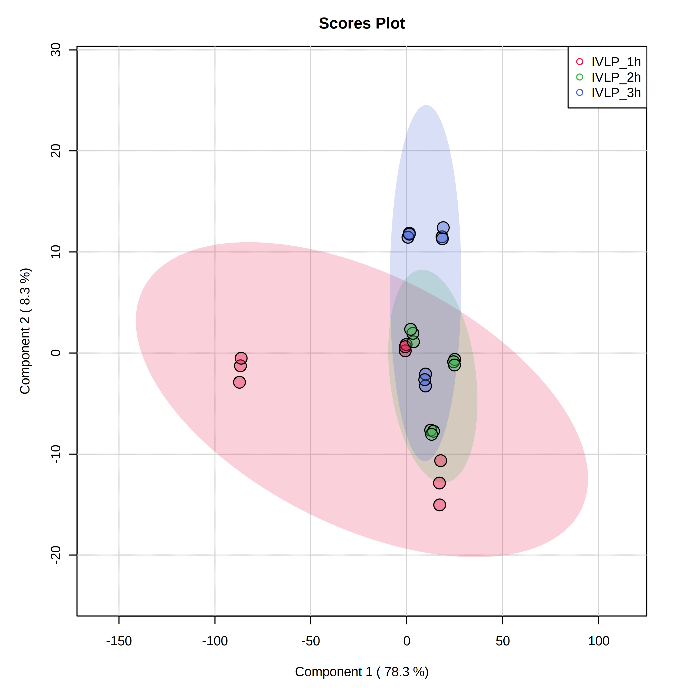


**C PL/MM/ESI- D**


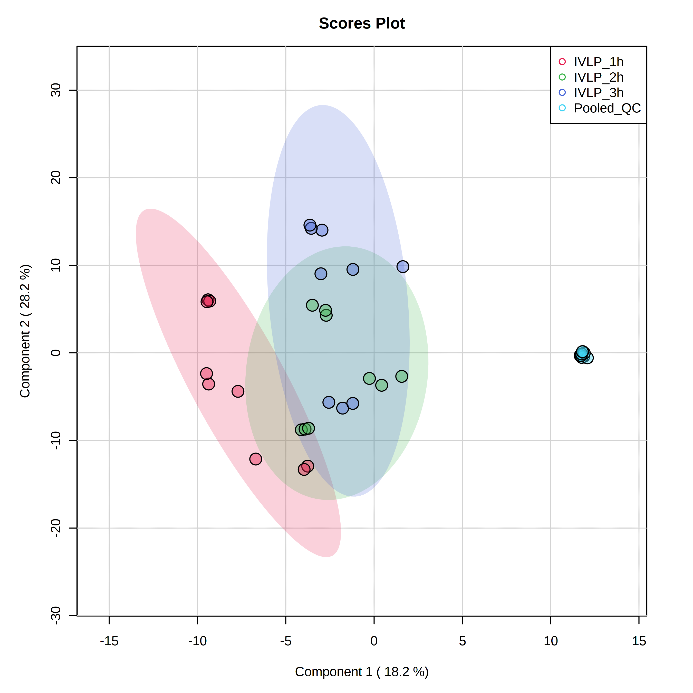

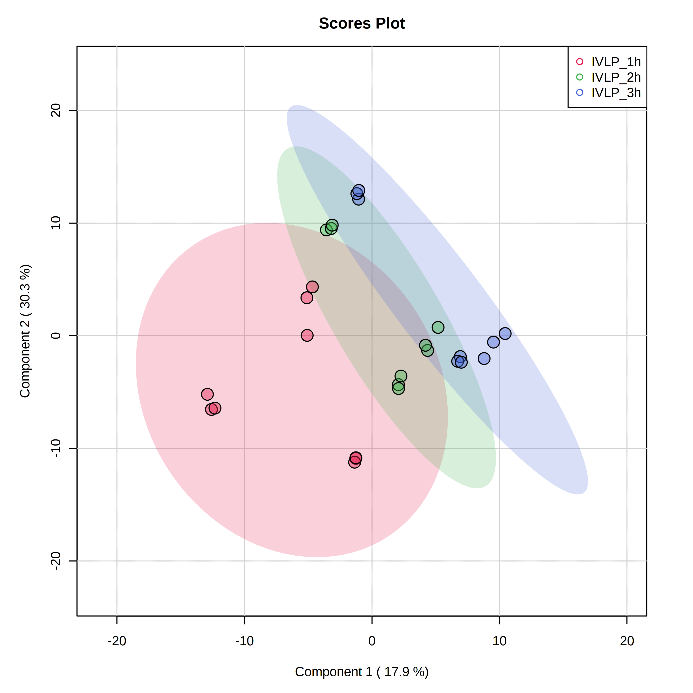


**Fig. S9.** *2D* *Partial least squares-discriminant analysis (PLS-DA) score plots for data obtained via (****A****,* ***B****) SPME–RP/LC–MS ESI (+) and (****C****,* ***D****) SPME–RP/LC–MS ESI (–) from plasma in porcine models when applying a low-dose regimen*. Abbreviations: **Pooled_QC**: pooled quality control samples; **IVLP_1h/2h/3h**: samples collected at the 1^st^, 2^nd^, and 3^rd^ hour of in vivo lung perfusion (IVLP); **PL**: plasma samples; **MM**: mixed-mode-based SPME coating; **ESI+**: positive ionization mode; **ESI–**: negative ionization mode.

**A**

**PFP/ESI+**

**B**

**C**

**D**

**PFP/ESI–**

**E**

**F**

**Fig. S10.** *Base peak chromatograms for selected lung tissue (****A****,* ***D****), perfusate (****B****,* ***E****), and plasma sample (****C****,* ***F****) collected in pentafluorophenyl (PFP)-based chromatographic mode.* **ESI+/−**: positive/negative ion mode.

**C18/ESI+**

**C18/ESI–**

**Fig. S11.** *Base peak chromatograms for a selected perfusate sample collected in C18-based chromatographic mode.* **ESI+/−**: positive/negative ion mode.

**Table S2.** *Metabolite levels significantly differing in the lung during in vivo lung chemo-perfusion*.

| Metabolite | HMDB/KEGG/  LIPID MAPS  Compound ID | Confidence  level | m/z | RT  [min] | Adduct | VIP score | Regulation  (up/down)* | Pathway |
| --- | --- | --- | --- | --- | --- | --- | --- | --- |
| Cyclic 6-hydroxymelatonin/  N-acetyltryptophan | HMDB60810/  HMDB13713 | 3  3 | 247.1077 | 13.67 | [M+H] ^+^ | 2.43 | ↑↓ | Oxidative stress/proteins’ stabilization |
| Lysophosphatidic acid (18:0)/ LPA(18:0) | HMDB07854 | 2/unique | 477.2381 | 17.21 | [M+K] ^+^ | 2.27 | ↑↓ | Intercellular lipid signaling |
| 7,10,13,16,19-Docosapentaynoic acid (FA 22:10)/  4,7,10,13,16-Docosapentaynoic acid (FA 22:10) | LMFA01030679  LMFA01030678 | 2  2 | 321.1848  321.1848 | 17.21  17.21 | [M+H] ^+^  [M+H] ^+^ | 2.18 | ↑↓ | Lipid metabolism |
| Octanoic acid | C06423 | 2 | 450.3788 | 19.46 | [3M+NH_4_] ^+^ | 1.98 | ↑↓ | Medium-chain FA oxidation |
| 3,11-Dioxopregna-4,17(20)-dien-21-oic acid methyl ester | C15267 | 3/unique | 357.2062 | 15.73 | [M+H] ^+^ | 1.97 | ↑↓ | Steroid metabolism |
| Resolvin D1 (RvD1)/  Resolvin D2 (RvD2) | HMDB03733/  HMDB02294 | 2 | 377.2323 | 14.81 | [M+H] ^+^ | 1.92 | ↑↓ | Resolution of inflammation |
| N-(15-tetracosenoyl)-sphing-4-enine | LMSP02010009 | 2/unique | 972.4428 | 17.22 | [3M+2H] ^2+^ | 1.89 | ↑↓ | Ceramide metabolism |
| N-Acetyl-galactosamine 4,6-disulfate | HMDB00843 | 2/unique | 594.4938 | 21.02 | [3M+2Na] ^2+^ | 1.88 | ↑↓ | Chondroitin sulfate synthesis |
| Cyclic phospatidic acid (18:0)/ CPA(18:0) | HMDB07004 | 2/unique | 459.2283 | 17.21 | [M+K] ^+^ | 1.85 | ↑↓ | Cellular signaling |
| Indoleacrylic acid | HMDB00734 | 2/unique | 188.0708 | 13.67 | [M+H] ^+^ | 1.69 | ↑↓ | Trp metabolism |
| Cytidine 3’-phosphate/  Cytidine 5’-phosphate | C05822/  C00055 | 2  2 | 324.0591  324.0591 | 12.06/  12.34 | [M+H] ^+^  [M+H] ^+^ | 1.66/  1.60 | ↑↓  ↑↓ | Purine metabolism  Purine metabolism |
| 5’-Methylthioadenosine | HMDB01173 | 2/unique | 298.0975 | 18.06 | [M+H] ^+^ | 1.60 | ↑↓ | Purine metabolism |
| Creatine | HMDB00064 | 2 | 132.0769 | 2.11 | [M+H] ^+^ | 1.56 | ↓↑ | ATP generation |
| 4-oxo-Retinoic acid | HMDB06285 | 2 | 315.1955 | 17.12 | [M+H] ^+^ | 1.49 | ↑↓ | Gap junctional communication in cells |
| Undecanoylcarnitine | HMDB13321 | 2 | 330.264 | 17.82 | [M+H] ^+^ | 1.44 | ↑↑ | FA metabolism |
| Palmitoylglycine | HMDB13034 | 2/unique | 314.2691 | 18.4 | [M+H] ^+^ | 1.35 | ↑↑ | FA metabolism |
| L-Glutamate | HMDB60475 | 2 | 148.0605 | 1.43 | [M+H] ^+^ | 1.33 | ↓↑ | AA metabolism |
| Carnosine | HMDB00033 | 2 | 227.1140 | 3.81 | [M+H] ^+^ | 1.29 | ↓↑ | Scavenging ROS |
| Citrulline | HMDB00904 | 2 | 176.1031 | 1.44 | [M+H] ^+^ | 1.27 | ↓↑ | NO generation |
| L-Tryptophan | HMDB00929 | 2 | 205.0973 | 13.67 | [M+H] ^+^ | 1.06 | ↑↓ | Trp metabolism |
| L-Arginine | HMDB00517 | 2/unique | 175.1191 | 2.37 | [M+H] ^+^ | 1.04 | ↓↑ | NO generation |
| L-Kynurenine | HMDB00684 | 2 | 209.0923 | 11.66 | [M+H] ^+^ | 1.01 | ↑↑ | Trp metabolism |
| 18-Hydroxycorticosterone | C01124 | 2 | 343.1915 | 15.77 | [M-H_2_O-H] ^−^ | 2.29 | ↑↓ | Aldosterone metabolism |
| Lipoxin A4/  Lipoxin B4 | HMDB04385/  HMDB05082 | 2  2 | 351.2178/ 351.2179 | 17.67/  16.64 | [M-H] ^−^ | 2.16/  1.84 | ↓↑ | Resolution of inflammation |
| Resolvin E2 (RvE2) | C18173 | 2 | 333.2074 | 17.67 | [M-H] ^−^ | 2.15 | ↓↑ | Resolution of inflammation |
| 11-Dehydrocorticosterone | C05490 | 3 | 343.1916 | 16.13 | [M-H] ^−^ | 1.90 | ↑↓ | Aldosterone metabolism |
| 19-Oxo-deoxy-corticosterone | HMDB12614 | 2 | 343.1915 | 17.16 | [M-H] ^−^ | 1.90 | ↑↓ | Aldosterone metabolism |
| 2-Hydroxylauroylcarnitine | HMDB13164 | 2/unique | 358.2601 | 17.11 | [M-H] ^−^ | 1.87 | ↑↑ | FA metabolism |
| Resolvin D1 (RvD1)/  Resolvin D2 (RvD2) | C18178/  C18179 | 2 | 375.2178 | 14.84 | [M-H] ^−^ | 1.81 | ↑↓ | Resolution of inflammation |
| Aldosterone | C01780 | 2 | 341.176 | 16.17 | [M-H_2_O-H] ^−^ | 1.78 | ↑↓ | Aldosterone metabolism |
| 5-Hydroxyindoleacetylglycine | C05832 | 2/unique | 247.0723 | 13.34 | [M-H] ^–^ | 1.78 | ↑↓ | AA metabolism |
| Ubiquinone-1 | HMDB02012 | 2 | 249.1132 | 19.31 | [M-H] ^−^ | 1.63 | ↑↓ | Mitochondrial oxidative stress |
| 18-Oxocortisol | HMDB00332 | 2 | 375.1815 | 15.77 | [M-H] ^–^ | 1.60 | ↑↓ | Cortisol metabolism |
| Acetylcholine | HMDB00895 | 2 | 145.1111 | 18.18 | [M-H] ^−^ | 1.47 | ↑↓ | Inflammation |
| Aminoadipic acid | HMDB00510 | 2 | 160.0608 | 1.49 | [M-H] ^−^ | 1.47 | →↑ | Lysine degradation |
| Prostaglandin E1 | HMDB01442 | 2 | 353.2336 | 17.25 | [M-H] ^−^ | 1.43 | ↓↑ | Vasodilation |
| Imidazol-5-yl-pyruvate | C03277 | 2 | 153.0298 | 14.26 | [M-H] ^–^ | 1.24 | ↑↓ | Histidine metabolism |
| 6-Keto-prostaglandin F1α | HMDB02886 | 2 | 369.2283 | 15.78 | [M-H] ^−^ | 1.10 | ↓↑ | Vasodilation |
| 9,10,13-TriHOME/  9,12,13-TriHOME | HMDB04710/  HMDB04708 | 2 | 329.2335/  329.2336 | 17.83/  16.94 | [M-H] ^−^ | 1.10/  1.07 | ↑↓ | Inflammation/  Cell signaling |
| Deoxycholic acid | HMDB00626 | 2 | 391.2856 | 18.92 | [M-H] ^−^ | 1.09 | ↑↓ | Lung injury/  inflammation |
| Arachidonic acid | HMDB01043 | 2 | 303.233 | 23.76 | [M-H] ^−^ | 1.01 | ↓↑ | Inflammation |
| Succinyl-L-proline | C11711 | 2/unique | 214.0718 | 9.72 | [M-H] ^−^ | 1.01 | →↑ | TCA cycle |

* The first arrow refers to the comparison of baseline vs. in vivo lung perfusion (IVLP), while the second refers to the comparison of IVLP vs. reperfusion.

Metabolites/lipids were organized according to the type of ionization mode and variable importance in projection (VIP) values.

**Table S3.** *Metabolite levels significantly differing in perfusate (MM probes) during lung chemo-perfusion*.

| Metabolite | HMDB/KEGG/  LIPID MAPS  Compound ID | Confidence  level | m/z | RT  [min] | Adduct | VIP score | Regulation (BL vs. IVLP) | Pathway |
| --- | --- | --- | --- | --- | --- | --- | --- | --- |
| 1-Methylnicotinamide | HMDB00699 | 2 | 236.5823 | 16.7 | [3M+Na+K] ^2+^ | 2.67 | ↓ | Nicotinate and nicotinamide metabolism |
| Dodecanedioylcarnitine | HMDB13327 | 2 | 452.1805 | 16.7 | [M+2K+H] ^3+^ | 2.59 | ↓ | FA metabolism |
| Hydroxycobalamin | HMDB02308 | 2 | 674.2966 | 18.7 | [M+2H] ^2+^ | 2.48 | ↓ | Storage/transport forms of vitamin B12 |
| Leukotriene C4 | HMDB01198 | 2 | 667.3363 | 18.7 | [M+ACN+H] ^+^ | 2.46 | ↓ | Inflammation/pulmonary fibrosis |
| 17-Hydroxyandrostane-3-glucuronide | HMDB10359 | 2 | 480.2703 | 16.69 | [2M+H+Na] ^2+^ | 2.43 | ↓ | Drug detoxification |
| Leukotriene E4 | HMDB02200 | 2 | 451.2368 | 16.69 | [2M+H+Na] ^2+^ | 2.38 | ↓ | Inflammation/pulmonary fibrosis |
| Androstenedione | HMDB00053 | 2 | 649.3064 | 18.7 | [2M+2K-H] ^+^ | 2.32 | ↓ | Androgen and estrogen metabolism |
| Glycochenodeoxycholate-3-sulfate | HMDB02497 | 2 | 494.2595 | 16.7 | [M+H-2H_2_O] ^+^ | 2.27 | ↓ | Apoptosis |
| Cyclic phosphatidic acid (18:1)/ CPA(18:1) | HMDB07006 | 2 | 666.333 | 18.7 | [3M+2K] ^2+^ | 2.27 | ↓ | Cellular signalling |
| Hydroxypropionylcarnitine | HMDB13125 | 2/unique | 234.1338 | 13.02 | [M+H] ^+^ | 2.17 | ↓ | Lipid peroxidation/FA oxidation |
| 2-Aminoadenosine | C00939 | 2/unique | 283.1153 | 13.32 | [M+H] ^+^ | 2.12 | ↓ | Purine metabolism |
| Glycerophosphocholine | HMDB00086 | 2/unique | 258.1102 | 1.13 | [M+H] ^+^ | 1.67 | ↓ | Lipid metabolism |
| Resolvin D1 (RvD1)/  Resolvin D2 (RvD2) | HMDB03733/  HMDB02294 | 3/  3 | 377.2323 | 14.81 | [M+H] ^+^ | 1.66 | ↑ | Resolution of inflammation |
| L-Octanoylcarnitine | HMDB00791 | 2 | 469.7789 | 13 | [3M+2K] ^2+^ | 1.38 | ↑ | FA metabolism |
| Taurocholic acid | HMDB00036 | 2 | 516.2989 | 13.28 | [M+H] ^+^ | 1.32 | ↑ | Lipid metabolism |
| L-Kynurenine | HMDB00684 | 2 | 209.0923 | 11.7 | [M+H] ^+^ | 1.27 | ↑ | Trp metabolism |
| Monoacylglycerol (24:6) | HMDB11590 | 2 | 431.3156 | 20.1 | [M+H] ^+^ | 1.17 | ↑ | Lipid metabolism |
| Sphingosine 1-phosphate | HMDB00277 | 2 | 380.256 | 19.6 | [M+H] ^+^ | 1.05 | ↑ | Lipid metabolism |
| L-Leucine | HMDB00687 | 2 | 132.102 | 8.85 | [M+H] ^+^ | 1.04 | ↑ | AA metabolism |
| L-Isoleucine | HMDB00172 | 2 | 132.102 | 7.84 | [M+H] ^+^ | 1.04 | ↑ | AA metabolism |
| L-Phenylalanine | HMDB00159 | 2 | 166.0864 | 11.11 | [M+H] ^+^ | 1.02 | ↑ | AA metabolism |
| 10,11-Dihydro-12R-hydroxy-leukotriene E4 | HMDB12501 | 2 | 469.2474 | 14.54 | [2M+H+Na] ^2+^ | 1.01 | ↑ | Inflammation/pulmonary fibrosis |
| Phosphatidylethanolamine (38:4)/ PE(38:4) | HMDB11448 | 2 | 752.5615 | 19.23 | [M-H] ^−^ | 2.71 | ↑ | Lipid metabolism |
| 2-Dodecenoylcarnitine | HMDB13326 | 2/unique | 681.5066 | 18.63 | [2M-H] ^−^ | 2.70 | ↑ | FA metabolism |
| Leukotriene D4 | C05951 | 2/unique | 743.3803 | 16.18 | [3M-2H] ^2−^ | 2.67 | ↑ | Inflammation |
| Dodecanedioic acid | C02678 | 2 | 689.446 | 17.94 | [3M-H] ^−^ | 2.59 | ↑ | FA metabolism/  ω-oxidation |
| Decanedioic acid | C08277 | 2 | 223.0949 | 17.61 | [M+Na-2H] ^−^ | 2.29 | ↑ | FA metabolism/ ω-oxidation |
| Resolvin D1 (RvD1)/  Resolvin D2 (RvD2) | HMDB03733/  HMDB02294 | 2/  2 | 375.2178 | 14.83 | [M-H] ^−^ | 2.28 | ↑ | Resolution of inflammation |
| Hydroxyoctanoic acid | HMDB00711 | 2 | 159.1019 | 14.58 | [M-H] ^−^ | 1.83 | ↑ | Lipid peroxidation/ FA metabolism |
| Thymidine | HMDB00273 | 2 | 241.0829 | 1.52 | [M-H] ^−^ | 1.72 | ↑ | Pyrimidine metabolism |
| Phosphatidylglycerolphosphate (34:2)/PGP(34:2) | HMDB13533 | 2 | 825.471 | 12.58 | [M-H] ^−^ | 1.70 | ↑ | Cardiolipin synthesis/Cell signaling |
| Aldosterone | C01780 | 2 | 341.1759 | 16.17 | [M-H_2_O-H] ^−^ | 1.69 | ↑ | Aldosterone metabolism |
| Retinoyl β-glucuronide | HMDB03141 | 2 | 475.2339 | 18.0 | [M-H] ^−^ | 1.60 | ↓ | Retinol metabolism |
| 4-oxo-Retinoic acid | HMDB06285 | 2 | 313.181 | 16.14 | [M-H] ^−^ | 1.26 | ↑ | Gap junctional communication in cells |
| N-15-tetracosenoyl-sphing-4-enine/ Cer(d18:1/24:1) | HMDB04953 | 2/unique | 970.429 | 16.12 | [3M-2H] ^2−^ | 1.14 | ↑ | Apoptosis |
| 9,10,13-TriHOME/  9,12,13-TriHOME | HMDB04710/  HMDB04708 | 2 | 329.2335 | 17.83 | [M-H] ^−^ | 1.11 | ↑ | Inflammation/  Cell signaling |
| Phosphatidylethanolamine (33:3)/ PE(33:3) | HMDB09153 | 2 | 698.4757 | 23.6 | [M-H] ^−^ | 1.10 | ↑ | Lipid metabolism |
| 5-Oxooctadecanoic acid | HMDB34074 | 2 | 297.2436 | 20.99 | [M-H] ^−^ | 1.09 | ↑ | FA metabolism |
| Phosphatidylcholine (28:2)/ PC(28:2) | HMDB07900 | 2/unique | 672.46 | 23.6 | [M-H] ^−^ | 1.08 | ↑ | Phosphatidylcholine biosynthesis |
| 11-Dehydrocorticosterone | HMDB04029 | 3 | 343.1913 | 16.13 | [M-H] ^−^ | 1.05 | ↑ | Aldosterone metabolism |
| Lysophosphatidylethanolamine (p-16:0)/LPE(p-16:0) | HMDB11152 | 2/unique | 436.2834 | 20.34 | [M-H] ^−^ | 1.05 | ↑ | Membrane phospholipid remodeling |
| 18-Hydroxycorticosterone | HMDB00319 | 2 | 343.1913 | 15.76 | [M-H_2_O-H] ^−^ | 1.04 | ↑ | Aldosterone metabolism |
| 18-Oxocortisol | HMDB00332 | 2 | 375.1814 | 15.76 | [M-H] ^−^ | 1.03 | ↑ | Cortisol metabolism |
| Ursocholanic acid | C19642 | 2/unique | 359.2962 | 25.35 | [M-H] ^−^ | 1.03 | ↑ | Membrane disruption |

Metabolites/lipids were organized according to the type of ionization mode and variable importance in projection (VIP) values.

**Table S4.** *Metabolites/lipids levels significantly differing in perfusate (C18 probes) during lung chemo-perfusion*.

| Metabolite/lipid | HMDB/KEGG/  LIPID MAPS  Compound ID | Confidence  level | m/z | RT  [min] | Adduct | VIP score | Regulation (BL vs. IVLP) | Pathway |
| --- | --- | --- | --- | --- | --- | --- | --- | --- |
| Propenoylcarnitine | HMDB13124 | 2 | 668.339 | 0.86 | [3M+Na] ^+^ | 2.70 | ↓ | Lipid peroxidation/FA oxidation |
| Glucosylceramide (d18:1/26:0) | HMDB04977 | 2 | 916.6384 | 10.17 | [M+2K-H] ^+^ | 2.46 | ↓ | Apoptosis/Cell signaling |
| Hydroxypropionylcarnitine | HMDB13125 | 2/unique | 234.1337 | 0.74 | [M+H] + | 2.02 | **↓** | Lipid peroxidation/FA oxidation |
| L-Octanoylcarnitine | HMDB00791 | 2/unique | 288.217 | 0.88 | [M+H] ^+^ | 1.40 | ↑ | FA oxidation |
| 9-Hexadecenoylcarnitine/  2-Hexadecenoylcarnitine | HMDB13207/  HMDB06317 | 2 | 812.673 | 9.89 | [2M+NH_4_] ^+^ | 1.38 | ↑ | FA oxidation |
| Ureidoisobutyric acid | HMDB02031 | 2 | 147.0764 | 0.62 | [M+H] ^+^ | 1.28 | ↑ | Pyrimidine metabolism |
| Tetradecanoylcarnitine | HMDB05066 | 2/unique | 372.3111 | 9.92 | [M+H] ^+^ | 1.21 | ↑ | FA oxidation |
| 7-Ketocholesterol | HMDB00501 | 2 | 401.3414 | 11.36 | [M+H] ^+^ | 1.16 | ↑ | Steroid metabolism |
| Ceramide phosphate (d18:1/26:1) | HMDB10707 | 2 | 811.6698 | 9.89 | [2M+3H_2_O+2H] ^2+^ | 1.16 | ↑ | Stimulation of arachidonic acid and prostanoid synthesis |
| Sphingomyelin (d17:1/24:1) | HMDB11696 | 2 | 811.6698 | 9.89 | [2M+H+Na] ^2+^ | 1.16 | ↑ | Cell signaling/apoptosis |
| Protoporphyrinogen IX | HMDB01097 | 2 | 569.3138 | 0.66 | [M+H] ^+^ | 1.05 | ↑ | Porphyrin metabolism (metabotoxin) |
| 18-Hydroxyretinoic acid | HMDB61095 | 2 | 344.2278 | 0.62 | [2M+3H_2_O+2H] ^2+^ | 1.05 | ↑ | Retinol metabolism |
| Resolvin D5 | HMDB04038 | 2 | 388.254 | 0.62 | [2M+3H_2_O+2H] ^2+^ | 1.01 | ↑ | Resolution of inflammation |
| Diacylglycerol (39:0) | HMDB55986 | 2 | 667.6214 | 20.68 | [M+H] ^+^ | 1.01 | ↑ | FA metabolism |
| Diacylglycerol (42:2) | HMDB56099 | 2 | 691.6217 | 20.46 | [M+H] ^+^ | 1.01 | ↑ | FA metabolism |
| Cholesteryl ester (20:5)/CE(20:5) | HMDB06731 | 2/unique | 671.5735 | 20.68 | [M+H] ^+^ | 1.01 | ↑ | Lipid metabolism |
| 12-Oxo-20-trihydroxy-leukotriene B4 | HMDB12553 | 2 | 383.2069 | 0.62 | [M+H] ^+^ | 1.01 | ↑↓* | Cell signaling/inflammation |
| Ceramide (d18:0/24:0) | HMDB11768 | 2 | 690.6181 | 20.46 | [M+K] ^+^ | 1.01 | ↑ | Cell signaling/apoptosis |
| Sphingomyelin (d18:0/16:1) | HMDB13464 | 2/unique | 703.5747 | 14.16 | [M+H] ^+^ | 1.01 | ↑ | Cell signaling/apoptosis |
| 3-Hydroxyoctanoic acid | HMDB01954 | 2 | 141.0913 | 1.88 | [M-H_2_O-H] ^–^ | 4.48 | ↑ | FA ω-oxidation |
| N-Octanoylglycine | HMDB00832 | 2 | 200.1287 | 1.11 | [M-H] ^–^ | 4.33 | ↑ | Medium-chain FA oxidation |
| L-Phenylalanine | HMDB00159 | 2 | 164.0709 | 0.76 | [M-H] ^–^ | 2.12 | ↑ | AA metabolism |
| Diacylglycerol (38:7)/DAG(38:7) | HMDB56368 | 2 | 637.4864 | 9.88 | [M-H] ^–^ | 1.85 | ↑ | Lipid metabolism |
| N-Oleoylglycine | HMDB13631 | 2/unique | 338.2703 | 9.97 | [M-H] ^–^ | 1.75 | ↑ | Stimulation of adipogenesis |
| Tetracosatetraenoic acid (24:4n-6) | HMDB06246 | 2 | 359.2958 | 12.9 | [M-H] ^–^ | 1.67 | ↑ | α-Linolenic acid and linoleic acid metabolism |
| 7,10-Hexadecadienoic acid | HMDB00477 | 2 | 251.2017 | 9.67 | [M-H] ^–^ | 1.66 | ↑ | Conjugated linoleic acid (CLA) metabolism |
| Dehydroepiandrosterone 3-glucuronide/  Dehydroisoandrosterone 3-glucuronide | HMDB10348  HMDB10327 | 2  2 | 463.2341  463.2341 | 0.87  0.87 | [M-H] ^–^  [M-H] ^–^ | 1.64  1.64 | ↑  ↑ | Excretion of toxic substances |
| Tetradecanedioic acid | HMDB00872 | 2 | 257.1759 | 5.16 | [M-H] ^–^ | 1.54 | ↑ | FA metabolism/ ω-oxidation |
| L-Histidine | HMDB00177 | 2 | 154.0614 | 1.59 | [M-H] ^–^ | 1.48 | ↑ | AA metabolism |
| Phosphatidylinositol (36:0) | HMDB09866 | 2 | 865.58 | 14.69 | [M-H] ^–^ | 1.47 | ↑ | Arachidonic acid biosynthesis |
| Adrenic acid/7,10,13,16-Docosatetraenoic acid | C16527 | 3 | 331.2644 | 11.95 | [M-H] ^–^ | 1.45 | ↑ | Prostacyclin inhibition/ prothrombotic activity |
| Lysophosphatidylcholine (17:0) | HMDB12108 | 2 | 508.3411 | 10.36 | [M-H] ^–^ | 1.39 | ↑ | Anti-inflammatory and vasoprotective activity |
| Docosa-4,7,10,13,16-pentaenoic acid/  Docosapentaenoic acid (22n-6) | HMDB60113 | 2 | 329.2487 | 11.34 | [M-H] ^–^ | 1.28 | ↑ | α-Linolenic acid and linoleic acid metabolism |
| Phosphatidylserine (38:0) | HMDB10164 | 2/unique | 818.5923 | 15.11 | [M-H] ^–^ | 1.26 | ↑ | Membrane phospholipid remodeling |
| Tetracosahexaenoic acid, n-3 | HMDB60117 | 2 | 355.2642 | 11.84 | [M-H] ^–^ | 1.26 | ↑ | Very-long-chain fatty acid metabolism |
| 4,8,12,15,19-Docosapentaenoic acid | HMDB39133 | 2 | 329.2486 | 11.62 | [M-H] ^–^ | 1.15 | ↓ | Very long-chain fatty acid metabolism |
| 5,8-Tetradecadienoic acid | HMDB00560 | 2 | 223.1701 | 8.18 | [M-H] ^–^ | 1.13 | ↑ | Unsaturated FA oxidation. |
| 11,14,17-Eicosatrienoic acid | C16522 | 2 | 305.2486 | 11.75 | [M-H] ^–^ | 1.12 | ↑ | Inhibition of fatty acid elongation/desaturation |
| Eicosadienoic acid | HMDB05060 | 2 | 307.2643 | 12.14 | [M-H] ^–^ | 1.05 | ↑ | Synthesis of n-6 eicosanoids |
| Tetracosapentaenoic acid (24:5n-3)/  Tetracosapentaenoic acid (24:5n-6) | HMDB06323  HMDB06322 | 2  2 | 357.2799  357.2799 | 12.41  12.41 | [M-H] ^–^  [M-H] ^–^ | 1.02  1.02 | ↑  ↑ | α-Linolenic acid and linoleic acid metabolism |

Metabolites/lipids were organized according to the type of ionization mode and variable importance in projection (VIP) values.
